# Supplementary material for: Neutrophils suppress tumor‐infiltrating T cells in colon cancer via matrix metalloproteinase‐mediated activation of TGFβ
Source: EMBO Mol Med. 2019 Dec 2;12(1):e10681. doi: 10.15252/emmm.201910681 (PMC6949488; doi:10.15252/emmm.201910681)
Supplement: Supplementary file 1 — Appendix [file EMMM-12-e10681-s001.pdf]

**Appendix**

**Table of contents**


- Appendix figure S1
- Appendix figure S2
- Appendix figure S3
- Appendix figure S4
- Appendix figure S5
- Appendix figure S6
- Appendix figure S7
- Appendix figure S8
- Appendix figure S9
- Appendix figure S10
- Appendix figure S11
- Appendix figure S12
- Appendix figure legends
- Appendix table S1
- Appendix table S2
- Appendix table S3
- Appendix table S4

Appendix Figure S1

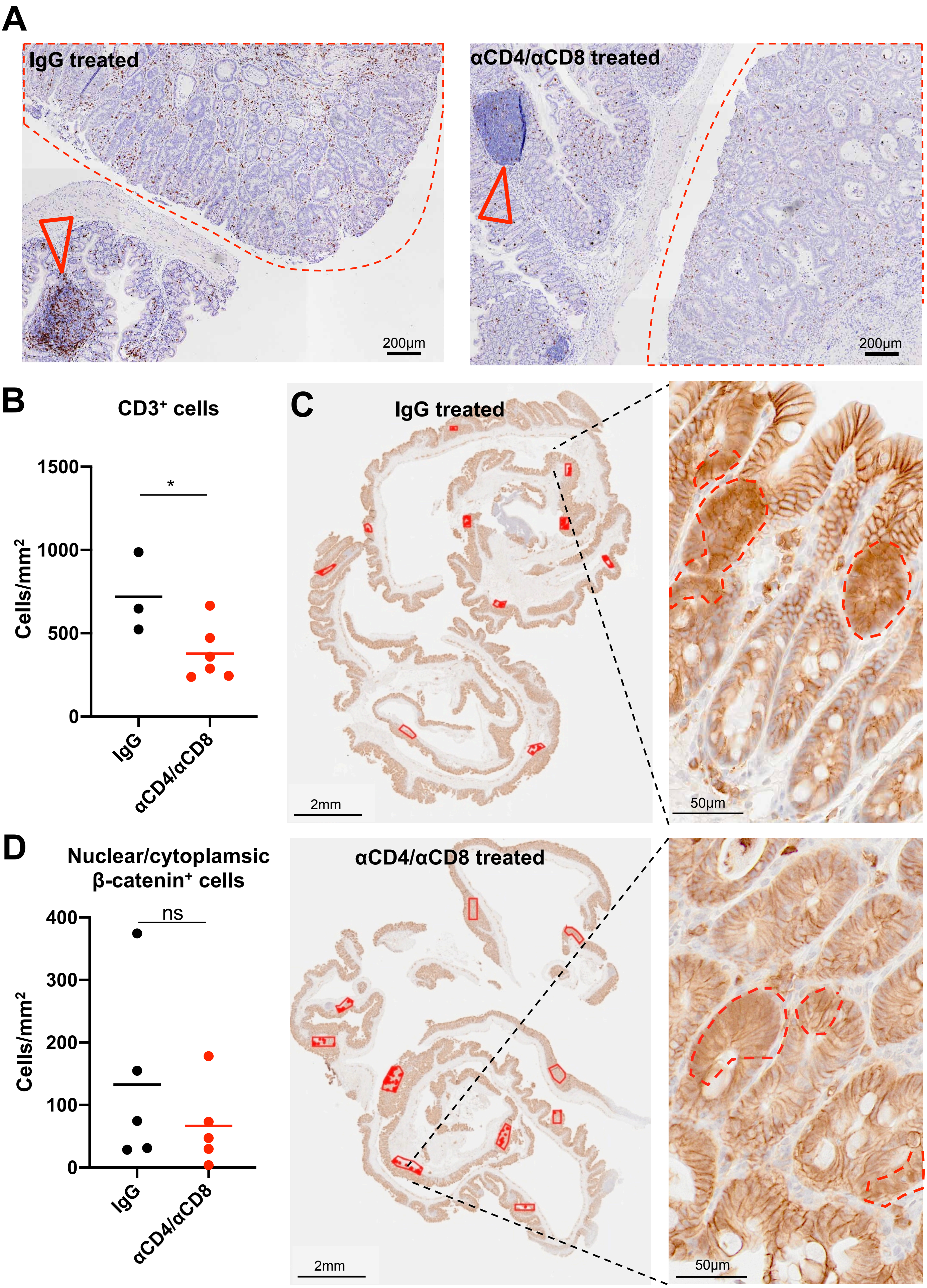

Appendix Figure S2

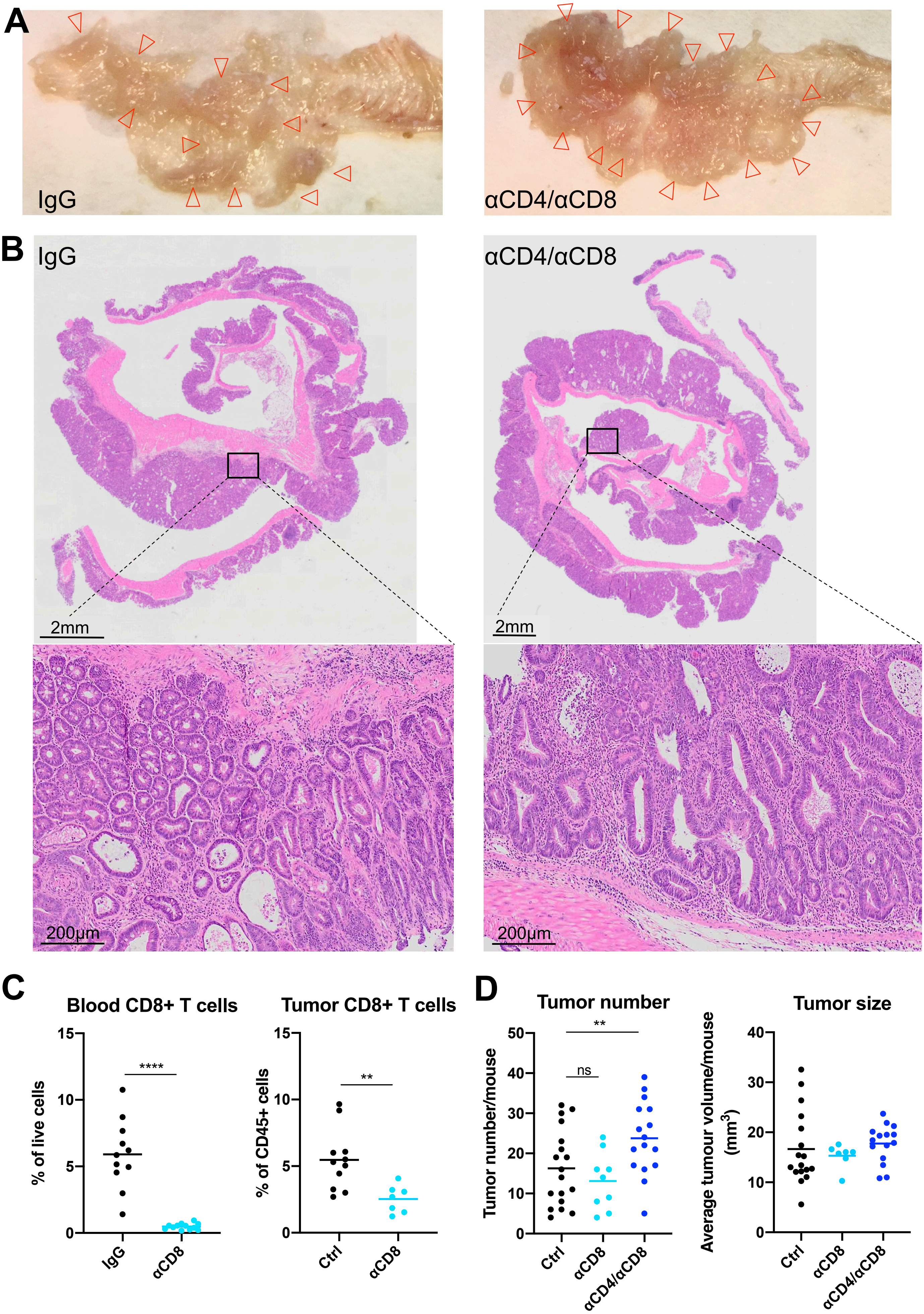

Appendix figure S3

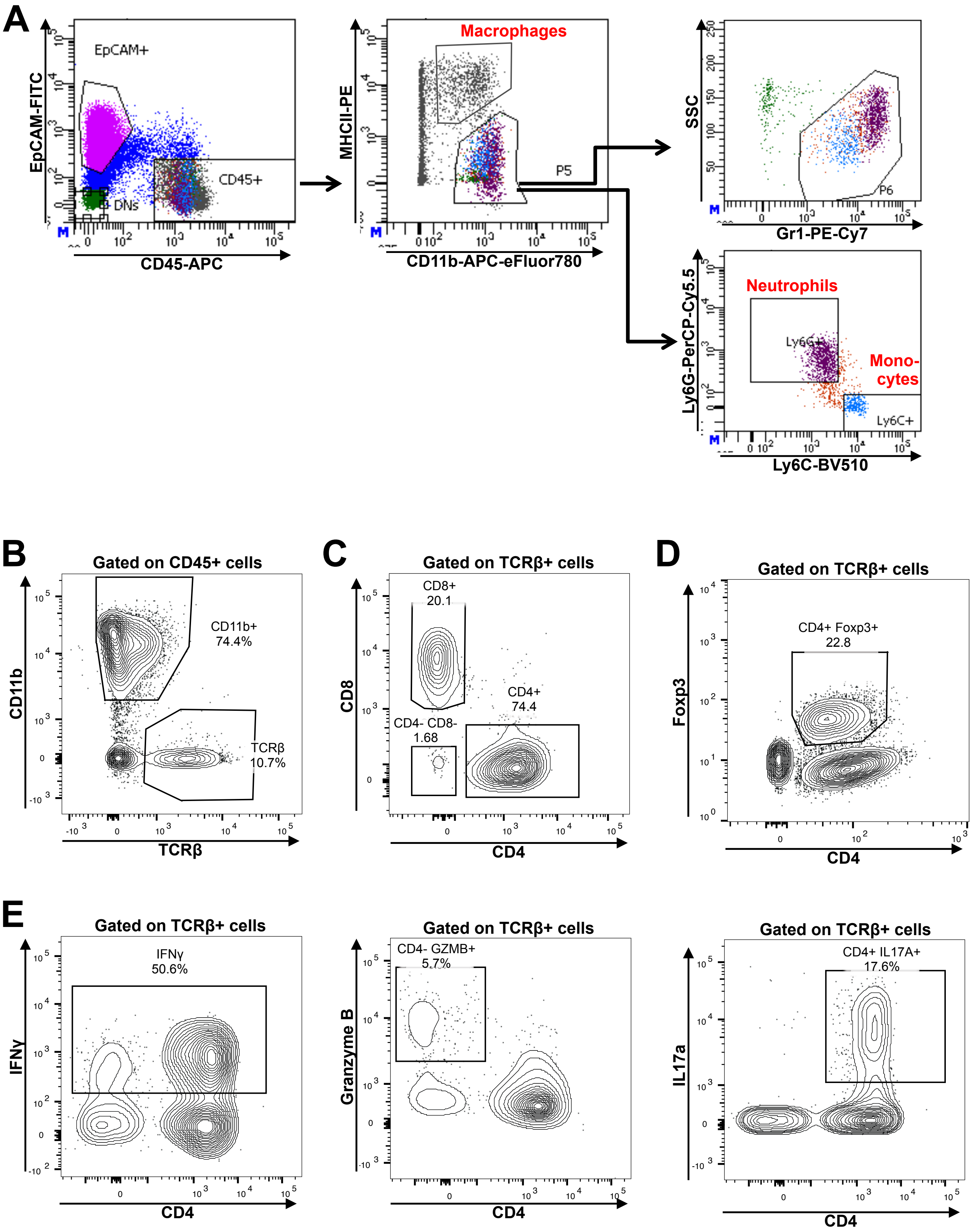

Appendix figure S4

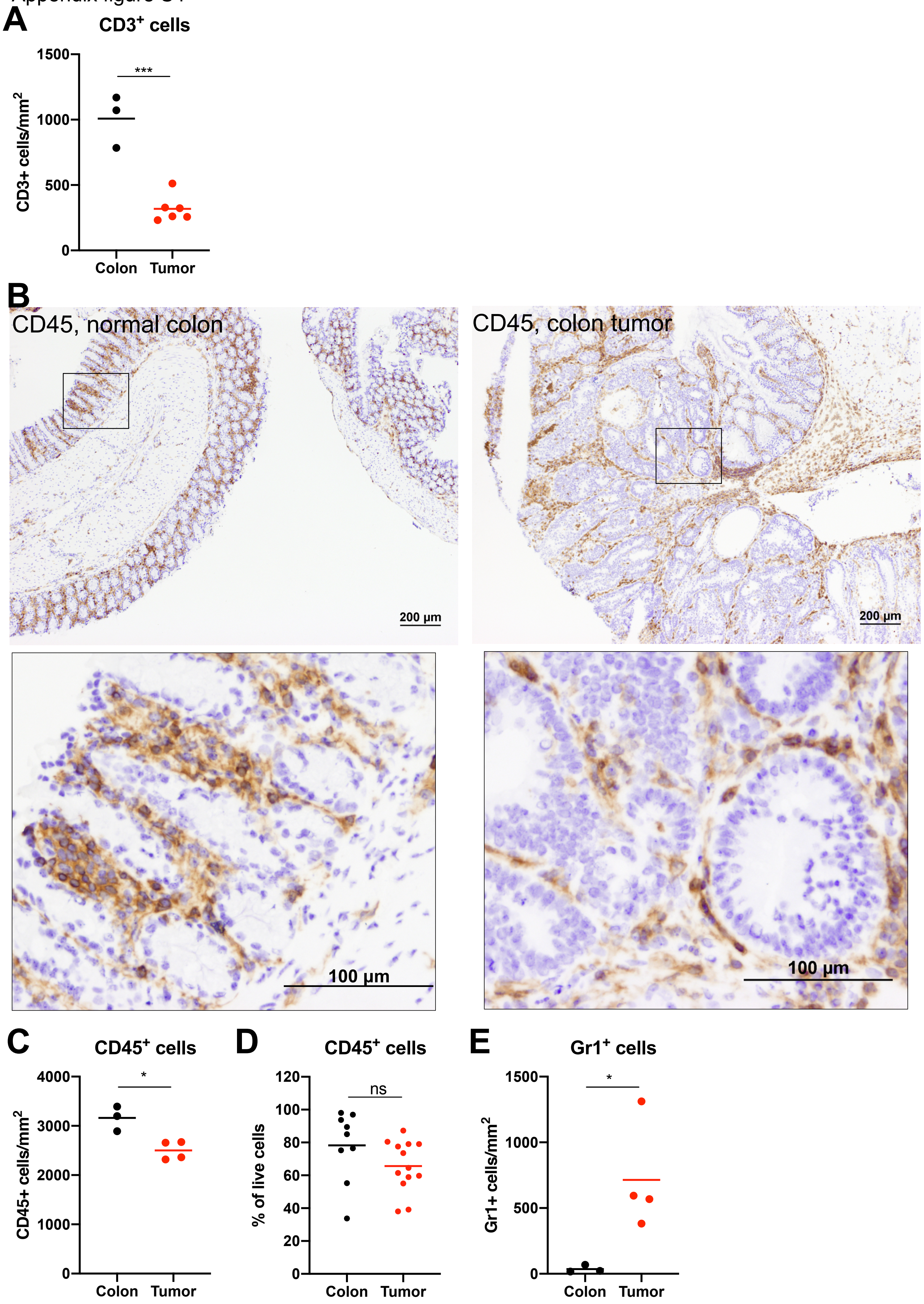

Appendix figure S5

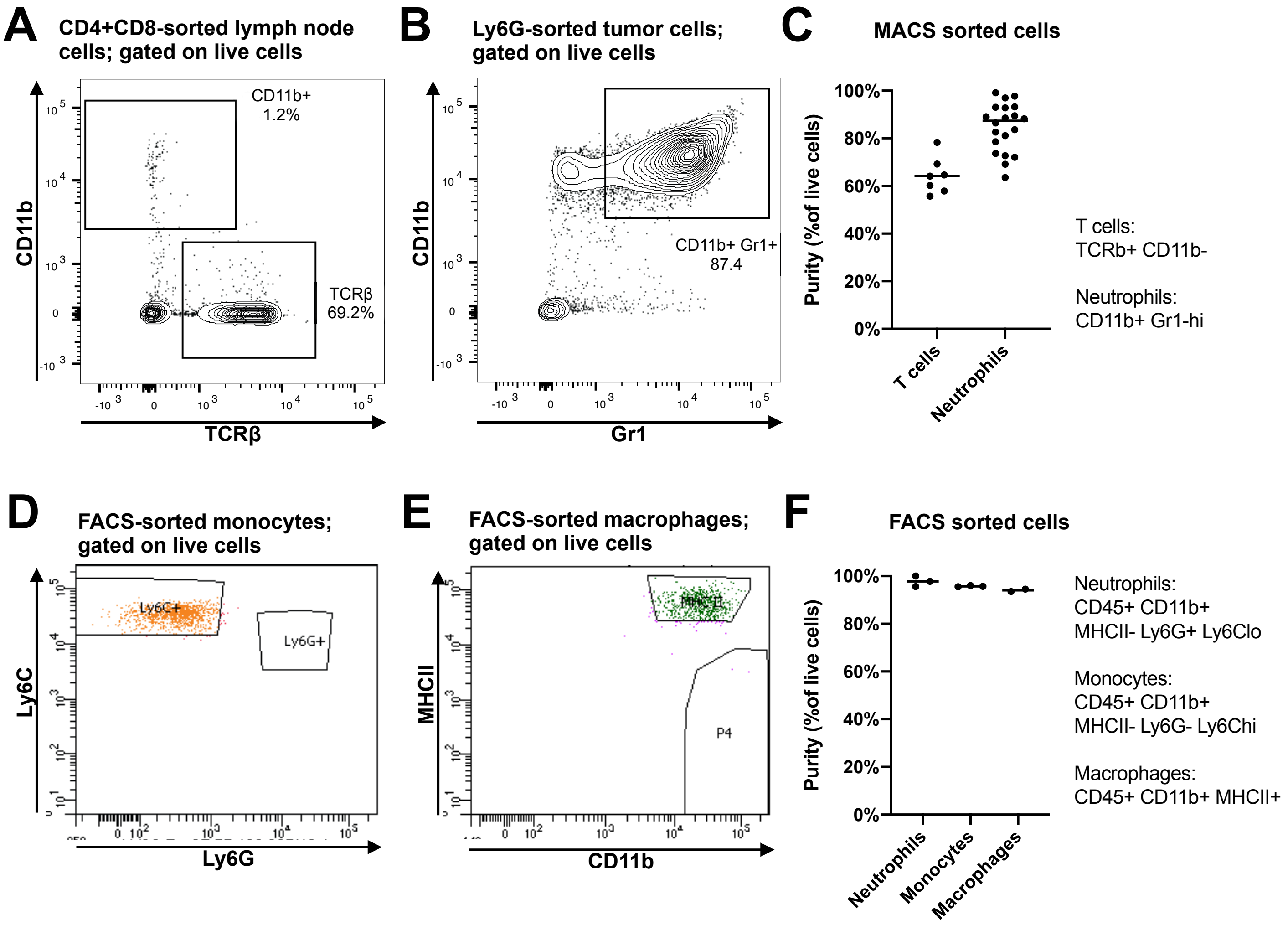

Appendix figure S6

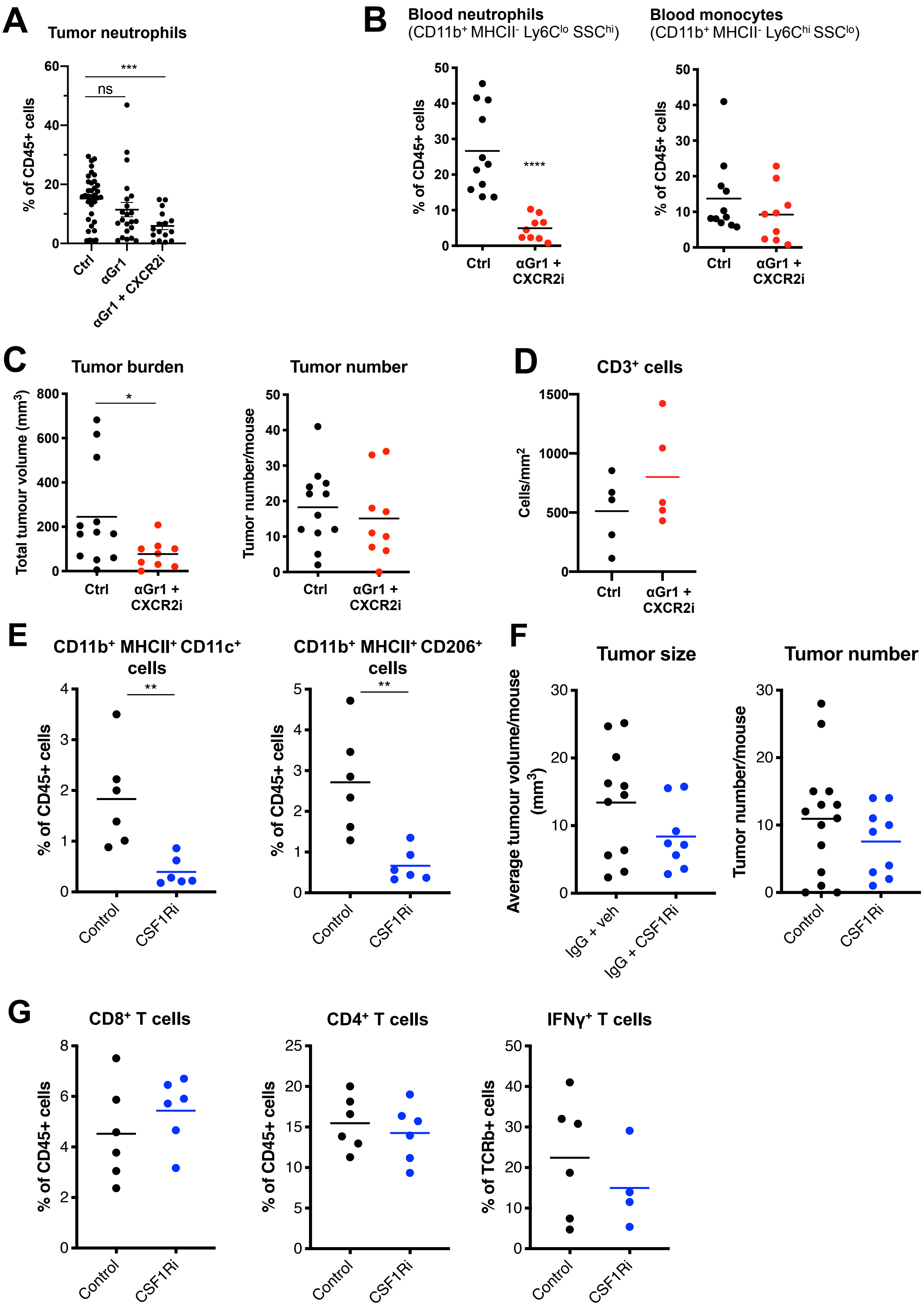

Appendix Figure S7

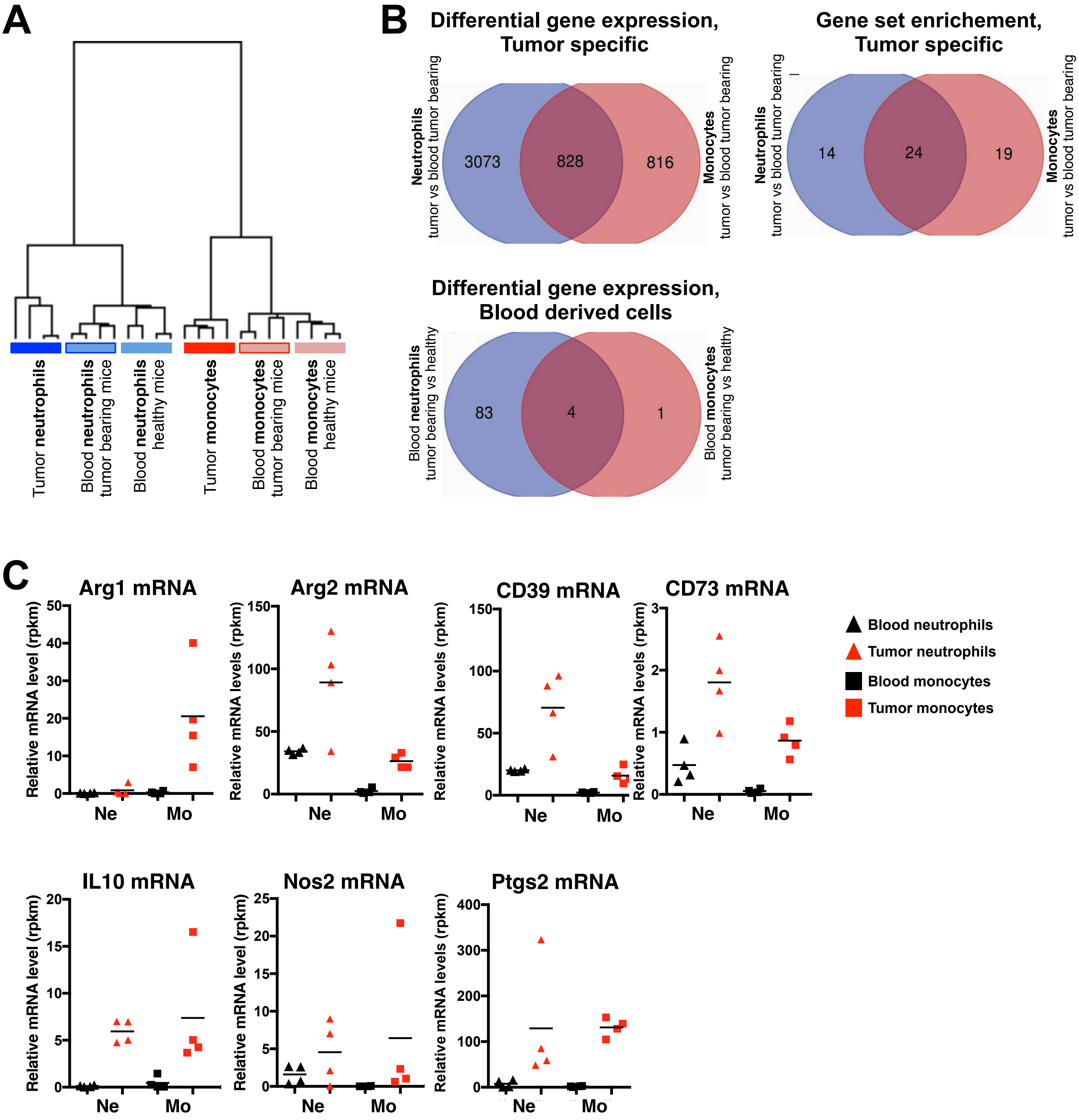

Appendix figure S8

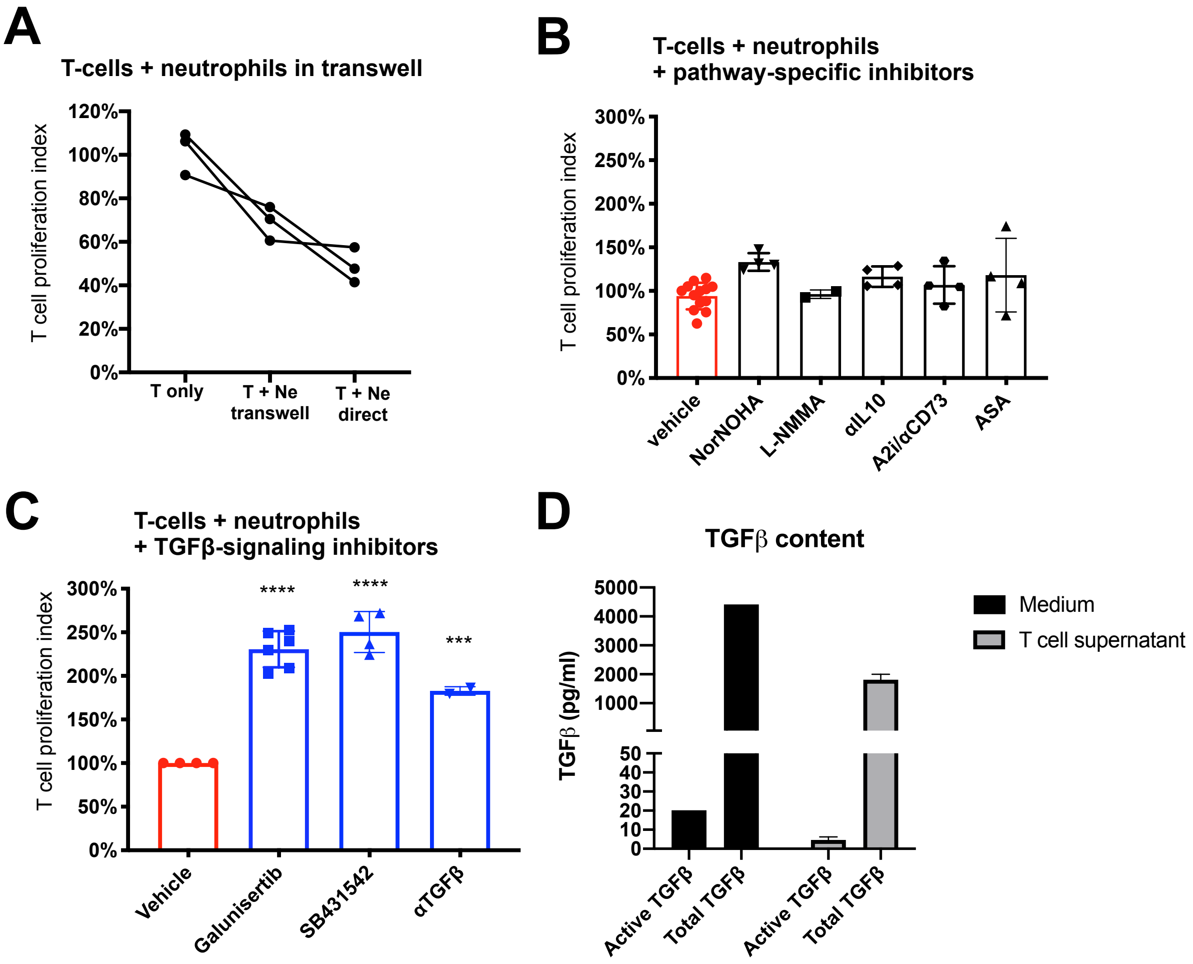

Appendix figure S9

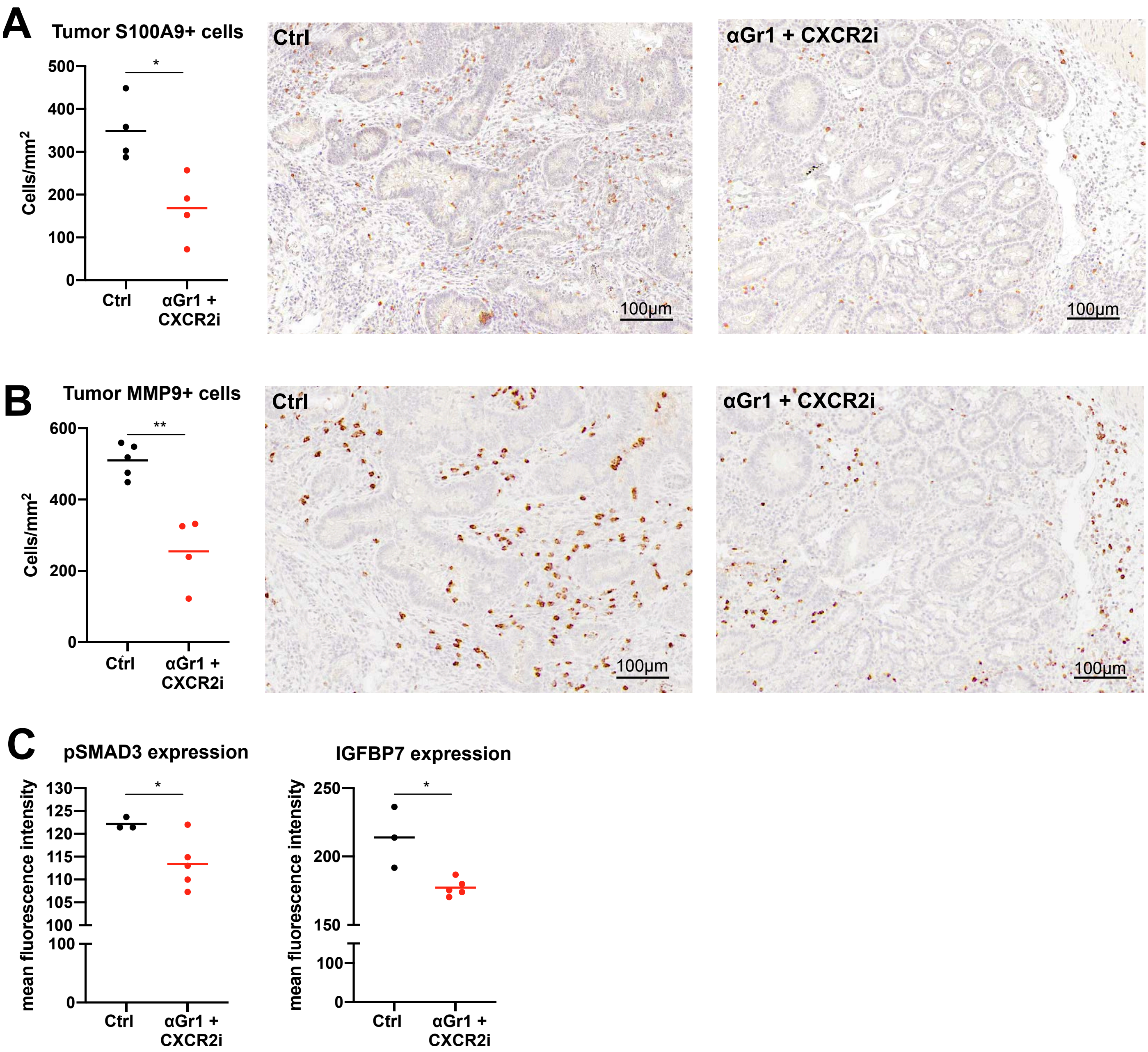

Appendix figure S10

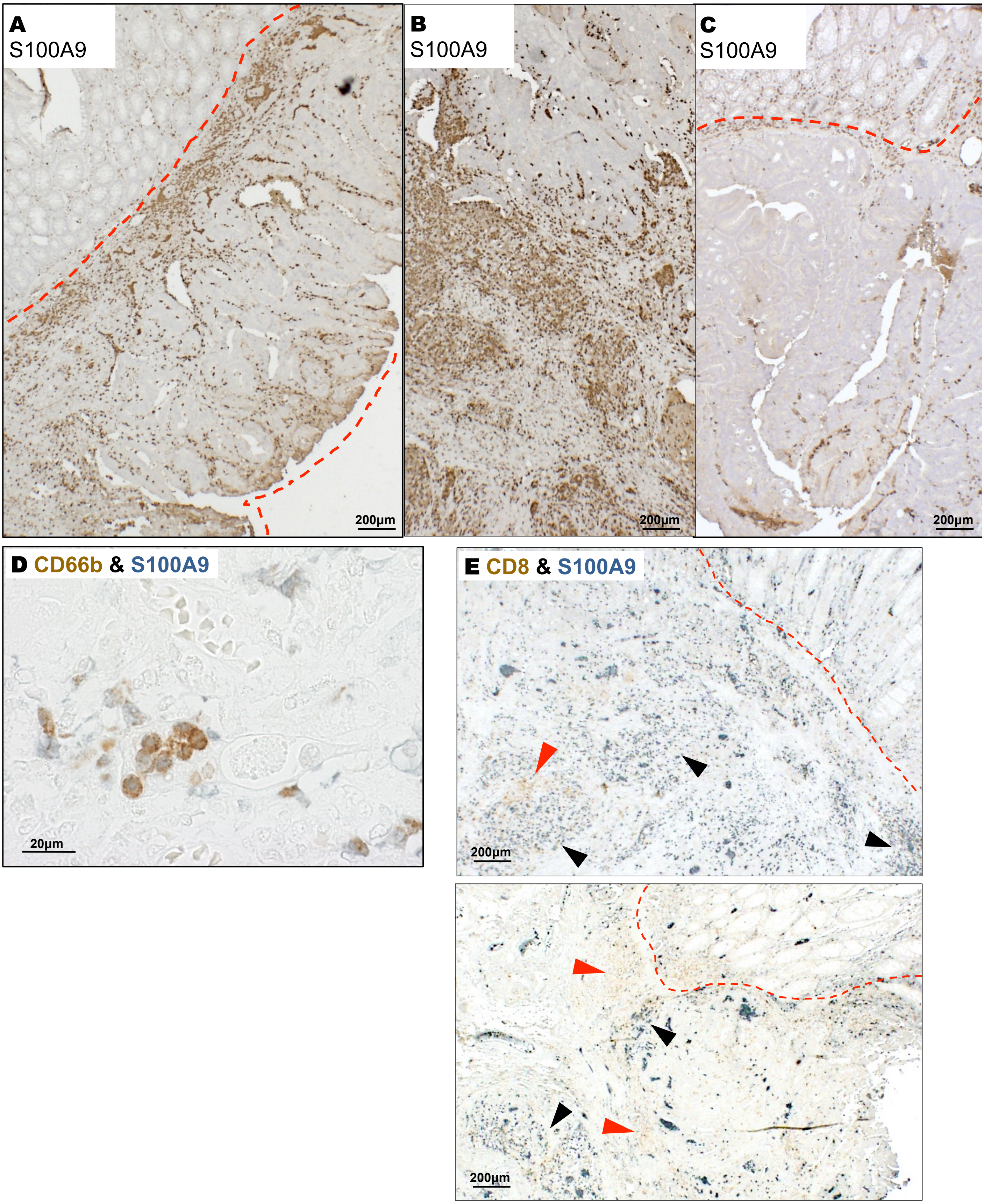

Appendix figure S11

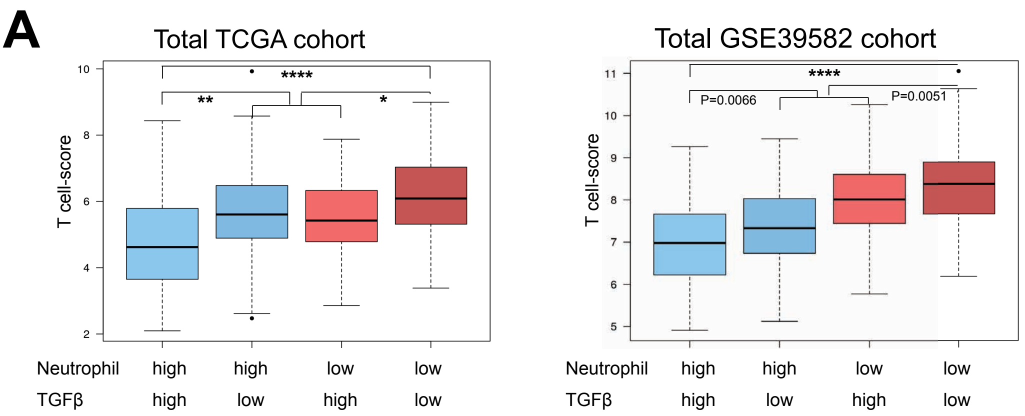


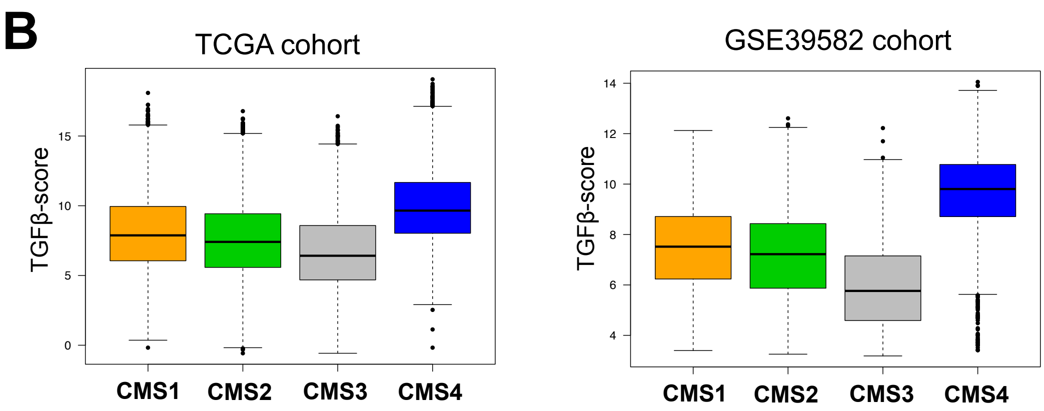

Appendix figure S12

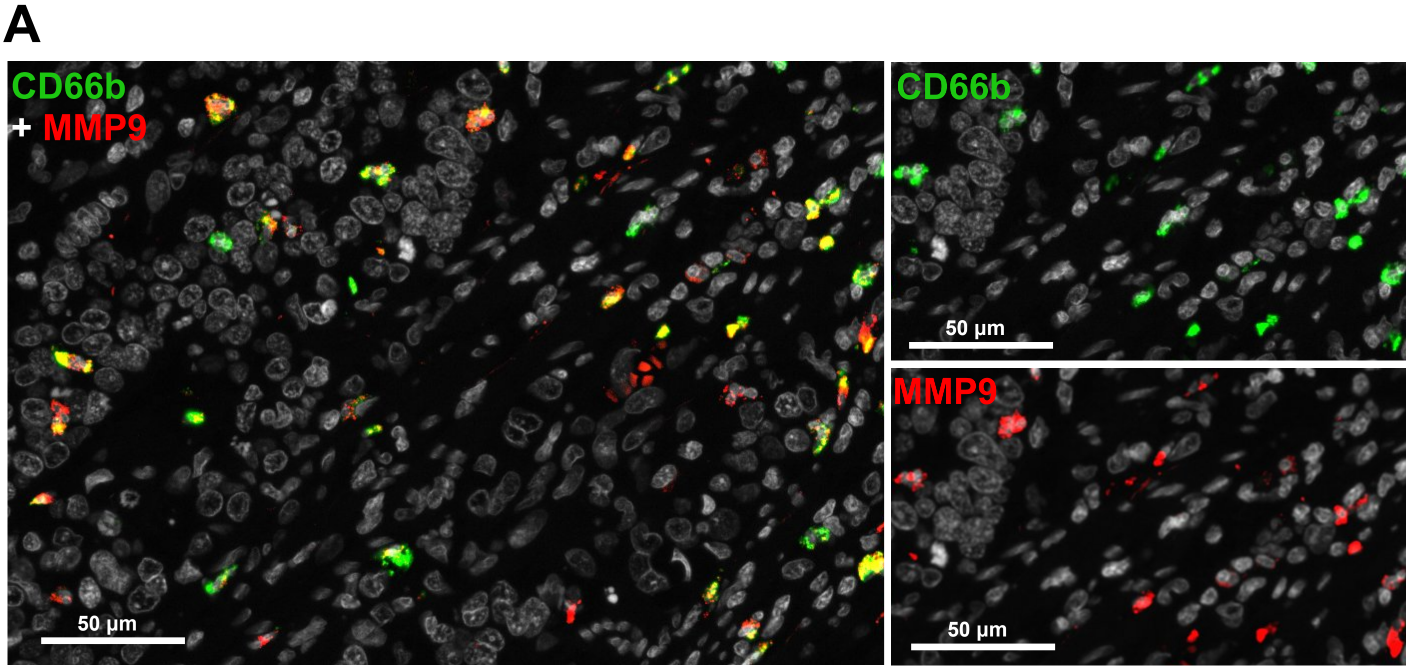


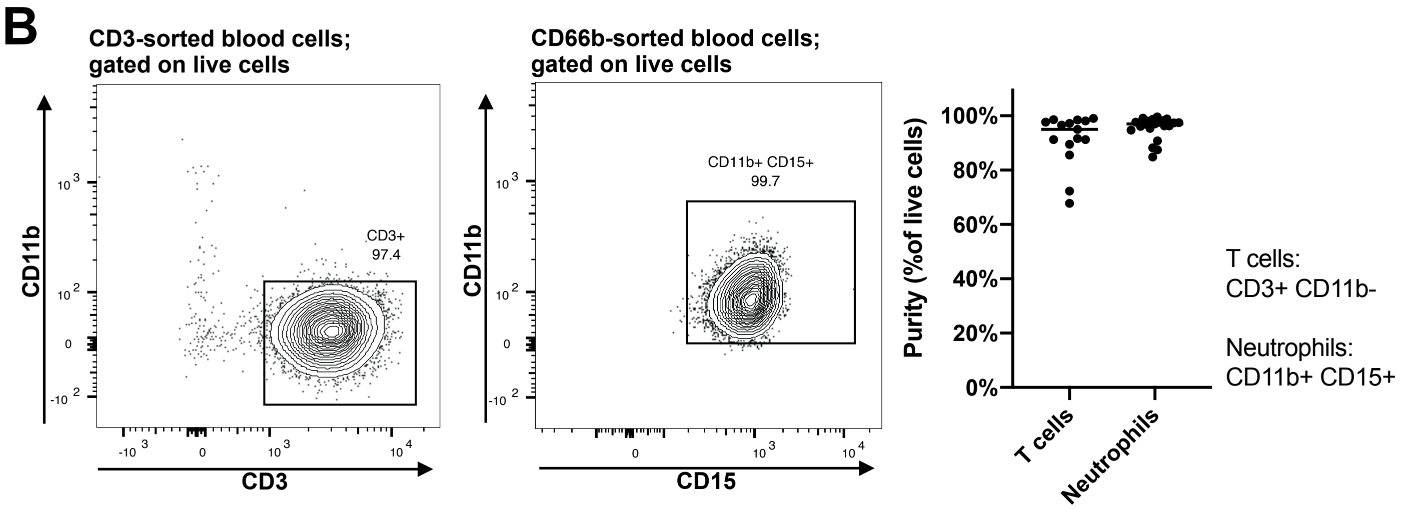

**Appendix figure legends**

**Figure S1. Effect of anti-CD4/anti-CD8 treatment on mouse colon adenoma T cell infiltration and on Tamoxifen-induced CreERT recombinase activity.**

A and B, *Apc*$^{fl/fl\text{-}Cdx2CreERT2}$ mice were treated with Tamoxifen and one day post treatment injected with anti-CD4 plus anti-CD8 neutralizing antibodies (αCD4/αCD8) or IgG control twice a week for six weeks. Colons were then excised and tissue sections were stained with anti-CD3 antibody and CD3$^{+}$ cells were quantified. A, Representative anti-CD3 immunostaining on sections derived from IgG treated (left panel) and αCD4/αCD8 treated (right panel) mice. Dashed lines outline tumor tissue. Arrowheads indicate tertiary lymphoid structures in adjacent benign colon mucosa. B, Quantification of CD3$^{+}$ cells in sections of tumors derived from IgG- (n=3) and αCD4/αCD8-treated (n=6) mice. At least five arbitrarily selected areas per section were scored.

C and D, *Apc*$^{fl/fl\text{-}Cdx2CreERT2}$ mice were treated with Tamoxifen and one day post treatment injected with anti-CD4 plus anti-CD8 neutralizing antibodies (αCD4/αCD8) or IgG control twice a week for five days. Colons were then excised and tissue sections were stained with anti-β-catenin antibody and cells with detectable nuclear β-catenin were quantified. C, Representative anti-β-catenin immunostaining on sections derived from IgG-(upper panel) and αCD4/αCD8-treated (lower panel) mice. Right panels are higher magnifications of indicated areas in left panels. Red delineated areas in left panels were the quantified areas on those sections. Red dashed lines in right panels outline cells with increased nuclear and cytoplasmic β-catenin staining. D, Quantification of nuclear and cytoplasmic β-catenin$^{+}$ cells in sections of tumors derived from IgG- (n=5) and αCD4/αCD8-treated (n=5) mice. Ten arbitrarily selected areas per section were scored.

Data information: In (B and D), statistical analysis was performed by unpaired two-tailed Student's t-tests. *: p<0.05; ***: p<0.001. Exact p-values are provided in Appendix table S4.

**Figure S2. Effect of anti-CD4/anti-CD8 treatment on mouse colon adenoma phenotype.**

*Apc*$^{fl/fl\text{-}Cdx2CreERT2}$ mice were treated with Tamoxifen and one day post treatment injected with anti-CD4 plus anti-CD8 neutralizing antibodies (αCD4/αCD8), anti-CD8 neutralizing antibody alone (αCD8) or IgG control twice a week for five to six weeks.

A, Dissected and cleaned cecum and proximal colon of *Apc*$^{fl/fl\text{-}Cdx2CreERT2}$ mice at the end of indicated treatments. Red arrowheads outline tumor containing tissue.

B, Cross sections of cecum and proximal colon of mice at the end of indicated treatments, stained with hematoxylin/eosin. Lower panels are higher magnifications of indicated areas in upper panels. Tumors in both IgG and αCD4/αCD8 treated mice displayed with an adenomatous, non-invasive phenotype.

C and D, Comparison of αCD8 treatment to αCD4/αCD8 treatment. C, Relative numbers of $CD8^+$ $TCR\beta^+$ cells determined by FACS in blood (left panel) and tumors (right panel) in mice at the end of indicated treatments (IgG: n=11; αCD8: n=7). D, Number (left panel) and average tumor size (right panel) per mouse at the end of indicated treatments (Pooled data from two independent experiments; IgG: n=18; αCD8: n=9; αCD4/αCD8: n=16).

Data information: In (C) and (D), each dot represents an individual mouse and exact group sizes are mentioned in brackets above. Statistical analysis was performed by

unpaired two-tailed Student's t-tests. **: $p<0.01$; ****: $p<0.0001$. Exact p-values are provided in Appendix table S4.

**Figure S3. FACS gating strategy to identify and sort individual myeloid cell and T cell populations.**

A, Gating strategy to identify and sort EpCAM$^{+}$ CD45$^{-}$ epithelial cells, EpCAM$^{-}$ CD45$^{-}$ stromal cells, CD45$^{+}$ CD11b$^{+}$ MHCII$^{+}$ macrophages, CD45$^{+}$ CD11b$^{+}$ MHCII$^{-}$ Ly6G$^{+}$ Ly6C$^{low}$ neutrophils and CD45$^{+}$ CD11b$^{+}$ MHCII$^{-}$ Ly6G$^{-}$ Ly6C$^{high}$ monocytes in tumor derived single cell suspension.

B, Gating strategy to identify TCRβ$^{+}$ cells among CD45$^{+}$ cells in tumor or blood derived single cell suspension.

C, Gating strategy to identify CD4$^{+}$ and CD8$^{+}$ T cell subsets among CD45$^{+}$ TCRβ$^{+}$ cells in tumor or blood derived single cell suspension.

D, Gating strategy to identify Foxp3$^{+}$CD4$^{+}$ regulatory T cells among CD45$^{+}$ TCRβ$^{+}$ cells in tumor derived single cell suspension.

E, Gating strategy to identify IFNγ$^{+}$ (left panel), CD4$^{-}$ Granzyme B$^{+}$ (middle panel) and CD4$^{+}$ IL17a$^{+}$ (right panel) effector T cell subsets among CD45$^{+}$ TCRβ$^{+}$ cells in tumor derived single cell suspension.

**Figure S4. Quantification of CD3$^{+}$ T cells, CD45$^{+}$ hematopoietic cells and Gr1$^{+}$ myeloid cells in mouse normal colon and colon adenomas by immunohistochemistry.**

Colons of healthy *Apc*$^{fl/fl}$ (Colon) or tumor bearing (Tumor) *Apc*$^{fl/fl\text{-}Cdx2CreERT2}$ mice 10-14 weeks after Tamoxifen treatment.

A, Quantification of $CD3^+$ cells using immunohistochemistry. Colon: n=3; Tumor: n=6.

B, Immunohistochemical staining for $CD45^+$ on representative sections of healthy normal colon (left panels) or colon tumor (right panels). Lower panels are higher magnifications of indicated areas in upper panels.

C, Quantification of $CD45^+$ cells using immunohistochemistry. Colon: n=3; Tumor: n=4.

D, Quantification of $CD45^+$ cells using FACS analysis described in Appendix Figure S3A. Colon: n=9; Tumor: n=13.

E, Quantification of $Gr1^+$ cells using immunohistochemistry. Colon: n=3; Tumor: n=4.

Data information: In (A) and (C-E), each dot represents an individual mouse. Statistical analysis was performed by unpaired two-tailed Student's t-tests. *: $p<0.05$; ***: $p<0.001$. Exact p-values are provided in Appendix table S4.

**Figure S5. Purity control of mouse T cells and neutrophils used for *in vitro* experiments.**

A - C, Purity of tumor-derived MACS sorted T cells and neutrophils used for *in vitro* co-culture assays**.** A, Representative purity test using TCRβ and CD11b FACS analysis on anti-CD4/anti-CD8 isolated lymph node cells. B, Representative purity test using Gr1 and CD11b FACS analysis on anti-Ly6G isolated tumor cells. C, Quantification of the purity of isolated $TCRb^+$ $CD11b^-$ cells and $CD11b^+$ $Gr1^+$ cells. Each dot represents an individually isolated sample. T cells: n=7; neutrophils: n=20.

D - F, Purity of tumor-derived FACS sorted neutrophils, monocytes and macrophages for *in vitro* co-culture assays and for cell type specific gene expression analysis. The gating strategy used is described in Appendix Fig. S3A. D,

Representative purity test on $CD45^+$ $CD11b^+$ $MHCII^-$ $Ly6G^-$ $Ly6C^{high}$ monocytes. E, Representative purity test on $CD45^+$ $CD11b^+$ $MHCII^+$ macrophages F, Quantification of the purity of isolated neutrophils, monocytes and macrophages. Each dot represents an individually isolated sample. Neutrophils: n=3; monocytes: n=3, macrophages: n=2.

**Figure S6. Effect of treatment of mice with anti-Gr1-neutralizing antibody, CXCR2-inhibitor or CSF1R-inhibitor on neutrophils, macrophages and colon tumor formation.**

A, Comparison of the effect of anti-Gr1-neutralizing antibody treatment (αGr1; n=22) to the combined αGr1 plus CXCR2-inhibitor (αGr1 + CXCR2i; n=16) on neutrophil tumor infiltration. Ctrl: n=35

B - D, Effect of αGr1 + CXCR2i treatment on blood neutrophil content and blood monocyte content determined by FACS analysis (B; Ctrl: n=11, aGr1 + CXCR2i: n=9), on total tumor burden and tumor number per mouse (C; Ctrl: n=12, aGr1 + CXCR2i: n=9) and on tumor $CD3^+$ cell content determined by immunohistochemistry (D; Ctrl: n=5, aGr1 + CXCR2i: n=5).

E - G, Effect of CSF1R-inhibitor (CSF1Ri) treatment on tumor M1 macrophages and M2 macrophages ($CD11b^+$ $MHCII^+$ $CD206^+$) content (E; Control: n=6, CSF1Ri: n=6**)**, on average tumor size and tumor number per mouse (F; Control: n=11, CSF1Ri: n=9) and on tumor $CD8^+$, $CD4^+$ and $IFN\gamma^+$ T cell content determined by FACS analysis (G; Control: n=6, CSF1Ri: n=6).

Data information: In (A – G), each dot represents an individual mouse and exact group sizes are mentioned in brackets above. Statistical analysis was performed by

unpaired two-tailed Student's t-tests. *: p<0.05; **: p<0.01; ***: p<0.001. Exact p-values are provided in Appendix table S4.

**Figure S7. Expression of T cell suppressive pathway genes in mouse tumor neutrophils and monocytes.**

RNA-sequencing analysis of neutrophils and monocytes isolated from colon tumors, blood of tumor-bearing mice or blood of healthy mice.

A, Unsupervised clustering of 1000 highest overall expressed genes.

B, Overlap of localization-specific gene expression pattern between neutrophils (blue) and monocytes (red). Differential gene expression was evaluated for tumor-derived cells compared to blood-derived cells (upper panels) and for cells derived from blood of tumor bearing mice compared to cells derived from blood of healthy mice (lower panel).

C, mRNA levels of indicated genes that have previously been described to be involved in myeloid cell mediated T cell suppression. Shown are mRNA levels in neutrophils (Ne; triangles) and monocytes (Mo; squares), derived from blood (black) or tumor (red), as reads per kilobase of transcript, per million mapped reads (rpkm); n=4 for all groups.

**Figure S8. *In vitro* activity of T cell-suppressive pathways in mouse tumor neutrophils and monocytes.**

*In vitro* co-culture of activated T cells with tumor-derived neutrophils.

A, Culture of T cells alone (T only), together with neutrophils plated in a transwell insert (T + Ne transwell) or together with neutrophils in direct contact (T & Ne direct).

Each line represents a culture established with neutrophils from an individual mouse. T cell proliferation index is numbers of proliferated T cells after three days of indicated co-culture condition relative to the number of proliferated T cells when cultured alone; n=3.

B and C, Proliferation of T cells in co-culture with neutrophils and treated with the arginase inhibitor NorNOHA (n=4), the iNOS inhibitor L-NMMA (n=2), an IL10 neutralizing antibody (αIL10; n=4), the adenosine receptor 2A inhibitor SCH58261 (A2i; n=4) and a CD73 neutralizing antibody (αCD73; n=4) or the Ptgs2 inhibitor acetylsalicylic acid (ASA; n=4), the TGFBR inhibitors Galunsertib (n=6) and SB431542 (n=4) or TGFβ neutralizing antibody (αTGFβ; n=2). T cell proliferation index is numbers of proliferated T cells after three days of indicated treatment as relative to the number of proliferated T cells when treated with vehicle.

D, Protein levels of free active TGFβ1 and of total TGFβ1 in T cell culture medium (n= 1) and T cell culture supernatants (n=4) determined by ELISA.

Data information: In (A – C), each dot represents a measurement on an individually derived sample and exact group sizes are mentioned in brackets above. In (C), statistical analysis performed by using one-way ANOVA with Dunnett's multiple comparison tests. ***: $p<0.001$; ****: $p<0.001$. Exact p-values are provided in Appendix table S4.

**Figure S9. Quantification of S100A9$^+$ cells, MMP9$^+$ cells and protein levels of pSMAD3$^+$ and IGFBP7$^+$ in tumors of neutrophil-depleted mice.**

Eight weeks after Tamoxifen treatment of *Apc*$^{fl/fl\text{-}Cdx2CreERT2}$ mice, animals were treated with anti-Gr1 antibody plus CXCR2 inhibitor (αGr1 + CXCR2i; n=4) or with IgG plus DMSO control (Ctrl; n=4) for one to three weeks. Tumor sections were then analyzed

for specific marker expression using immunohistochemistry (A and B) and immunofluorescence (C).

A, Quantification (left panel) and immunostaining of representative tumor sections (middle and right panel) for S100A9+ cells.

B, Quantification (left panel) and immunostaining of representative tumor sections (middle and right panel) for $MMP9^+$ cells. Ctrl: n=5, $\alpha$Gr1 + CXCR2i: n=4.

C, Quantification of pSMAD3 (left panel) and IGFBP7 (right panel) staining intensity per positive cell. Each dot represents an individual mouse. Ctrl: n=3, $\alpha$Gr1 + CXCR2i: n=5.

Data information: In (A – C), at least five arbitrarily selected areas per section were scored. Statistical analysis was performed by unpaired two-tailed Student's t-tests. *: $p<0.05$; **: $p<0.01$. Exact p-values are provided in Appendix table S4.

**Figure S10. Neutrophil infiltration in human colon tumors.**

Immunostainings for neutrophils and $CD8^+$ T cells on representative human CRC sections.

A - C, Anti-S100A9 immunostainings described in table S1. A, Sample with high $S100A9^+$ cell infiltration at tumor border and in tumor center. B, Sample with high $S100A9^+$ cell infiltration in tumor center. C, Sample with high $S100A9^+$ cell infiltration at tumor border and in benign tissue, but not in tumor center.

D, Co-localization of immunostainings for neutrophil markers S100A9 (brown) and CD66b (grey-blue).

E, Section co-stained for the T cell marker CD8 (brown) and the neutrophil marker S100A9 (grey-blue). Upper and lower panels depict two areas on the same section.

Black arrowheads indicate foci of S100A9$^+$ cells and red arrowheads indicate foci of CD8$^+$ cells.

A – C and E, Red dashed lines indicate tumor border.

**Figure S11. Comparison of gene expression signatures indicative of T cells, neutrophils and TGFβ signaling in CRC and colon adenoma datasets.**

Gene expression signatures and scores specific for either T cells, TGFβ signaling or neutrophils were generated as described in material and methods.

A, T cell score in categories of CRC samples according to neutrophil and TGFβ scores.

B, TGFβ scores in categorization of CRC samples according CMS subtypes. Boxes are lower and upper quartiles with median as solid lines; horizontal lines define minimum and maximum; dots define outliers.

Data information: In (A), p-values were calculated with Mann-Whitney test. *: $p<1x10^{-3}$; **: $p<1x10^{-6}$; ***: $p<1x10^{-8}$; **** $p<1x10^{-10}$. Exact p-values are provided in Appendix table S4.

**Figure S12. MMP9 expression by human neutrophils and purity of isolated human neutrophils and T cells.**

A, Co-immunostaining for MMP9 (red) and CD66b (green) with DAPI counterstaining on a representative human CRC section. Left panel is merged channels for CD66b- and MMP9-specific fluorescent staining. Right panels show a region of left panel with individual channels for CD66b- and MMP9-specific fluorescence.

B, Purity of CRC patient and healthy volunteer derived blood T cells and neutrophils used for in vitro co-culture assays. T cell purity was tested with CD3 plus CD11b FACS analysis on anti-CD3 MACS-isolated cells. Neutrophil purity was tested using CD11b plus CD15 FACS analysis on anti-CD66b MACS isolated cells. Each dot represents an individual human sample. T cells: n=15; Neutrophils: n=21.

**Appendix Table S1**

**Information on cell marker panels used to analyze and sort individual cell types**

| **Species** | **Cell type** | **Marker MACS** | **Marker FACS** |
|---|---|---|---|
| Mouse | T cell | CD4 & CD8 | CD45+ EpCAM- TCRb+ |
| Mouse | Neutrophil | Ly6G | CD45+ EpCAM- CD11b+ MHCII- Ly6C-low Ly6G+ |
| Mouse | Monocyte | not done | CD45+ EpCAM- CD11b+ MHCII- Ly6C-high Ly6G- |
| Mouse | Macrophage | not done | CD45+ EpCAM- CD11b+ MHCII+ |
| Mouse | Epithelial cell | not done | CD45- EpCAM+ |
| Mouse | Stromal cell | not done | CD45- EpCAM- |
| | | | |
| Human | T cell | CD3 | CD45+ CD3+ |
| Human | Neutrophil | CD66b | CD45+ CD11b+ HLA-DR- CD14- CD15+ CD66b+ |

**Appendix Table S2**

Gene signatures indicative for the presence of neutrophils, T cells or TGFβ signaling. Individual gene signatures for TCGA and GSE39582 were generated as described in Material and Methods

| **Cohort** | TCGA | | | | | |
|---|---|---|---|---|---|---|
| **Samples** | CMS1-4 | | | CMS4 | | |
| **Signature type** | Neutrophils | T cells | TGFβ | Neutrophils | T cells | TGFβ |
| **Signature genes** | AQP9 | CCL5 | ADAM12 | AQP9 | ABCD2 | ADAM12 |
| | CSF3R | CD2 | ADAMTS16 | CXCL5 | CCL5 | ADAMTS16 |
| | CXCL5 | CD27 | AEBP1 | CXCR1 | CD2 | AEBP1 |
| | CXCR1 | CD3D | ANTXR1 | CXCR2 | CD226 | APCDD1L |
| | CXCR2 | CD3E | AOC3 | FCAR | CD27 | ARSI |
| | FCAR | CD3G | ASPN | FCGR3B | CD38 | C1QTNF3 |
| | FCGR3B | CD7 | BGN | FPR2 | CD3D | C5orf46 |
| | FPR2 | CD8A | BNC2 | IL1B | CD3E | CALB2 |
| | PROK2 | CD96 | CCDC8 | IL24 | CD3G | CERCAM |
| | S100A8 | CIITA | CCDC80 | MEFV | CD40LG | CHST6 |
| | | CRTAM | COL10A1 | MMP1 | CD8A | CIDEC |
| | | CTSW | COL11A1 | MMP3 | CD96 | CILP |
| | | CXCL9 | COL1A1 | PROK2 | CXCL9 | CNTN1 |
| | | CXCR6 | COL1A2 | S100A12 | CXCR2P1 | COL1A1 |
| | | FASLG | COL3A1 | S100A8 | CXCR6 | COL1A2 |
| | | FCRL6 | COL8A1 | SERPINB2 | EOMES | COL3A1 |
| | | GBP4 | COL8A2 | | FASLG | COMP |
| | | GBP5 | CTHRC1 | | FCRL5 | CPXM1 |
| | | GNLY | CYS1 | | GBP4 | EPYC |
| | | GPR171 | EFEMP1 | | GBP5 | FABP4 |
| | | GPR174 | EVC | | GZMA | FBLN2 |
| | | GZMA | FBLN1 | | GZMH | FNDC1 |
| | | GZMH | FBLN2 | | GZMK | FST |
| | | GZMK | FBN1 | | IRF4 | GAS1 |
| | | HLA-DOA | FIBIN | | ITK | GDF6 |
| | | IDO1 | FNDC1 | | JAKMIP1 | GPIHBP1 |
| | | IFNG | GLI3 | | KLRB1 | HTRA1 |
| | | IL12RB1 | HMCN1 | | KLRK1 | IGFL1 |
| | | IL21R | HTRA3 | | LAX1 | IGFL2 |
| | | IL2RB | ISLR | | LY9 | KLK5 |
| | | ITGAL | ITGA11 | | MAP4K1 | MFAP5 |
| | | ITK | ITGBL1 | | NKG7 | MMP23A |
| | | JAKMIP1 | LUM | | P2RY10 | NPR3 |
| | | KLRK1 | MRGPRF | | PDCD1 | OMD |
| | | LAG3 | MSRB3 | | PLA2G2D | PCDHGB5 |
| | | MAP4K1 | NAP1L3 | | PTPRCAP | PDGFRL |
| | | NKG7 | NTM | | PYHIN1 | PTH1R |
| | | P2RY10 | P4HA3 | | RASGRP1 | SCARF2 |
| | | PDCD1 | PDLIM3 | | SH2D1A | SFRP1 |
| | | PYHIN1 | PTGER3 | | SIRPG | SFRP2 |
| | | SH2D1A | SCARF2 | | SLA2 | SFRP4 |
| | | SIRPG | SFRP4 | | SLAMF1 | SNCG |
| | | SLA2 | SGCD | | SLAMF7 | SPON1 |
| | | SLAMF7 | SPOCK1 | | TBX21 | SSC5D |
| | | TBX21 | SSC5D | | THEMIS | TGFB3 |
| | | THEMIS | ST6GALNAC5 | | TIGIT | THBS2 |
| | | TIGIT | SULF1 | | TRAT1 | WISP2 |
| | | TRAT1 | TAGLN | | UBASH3A | |
| | | UBASH3A | THBS2 | | | |
| | | ZNF683 | | | | |

| **Cohort** | GSE39582 | | | | | |
|---|---|---|---|---|---|---|
| **Samples** | CMS1-4 | | | CMS4 | | |
| **Signature type** | Neutrophils | T cells | TGFβ | Neutrophils | T cells | TGFβ |
| **Signature genes** | AQP9 | APOBEC3G | ACTG2 | AQP9 | AIM2 | ADAM12 |
| | BCL2A1 | C1QA | ANTXR1 | BCL2A1 | APOBEC3G | ASPN |
| | CCL3 | C1QB | ASPN | CCL3 | BTN3A3 | COL10A1 |
| | CHI3L1 | CCL5 | COL10A1 | CHI3L1 | C1QB | COL11A1 |
| | CXCL1 | CCR5 | COL11A1 | CXCL1 | CCL5 | COL12A1 |
| | CXCL2 | CD2 | COL1A1 | CXCL3 | CCR5 | COL1A1 |
| | CXCL3 | CD247 | COL1A2 | CXCL5 | CD2 | COL1A2 |
| | CXCL5 | CD27 | COL8A1 | CXCL6 | CD247 | COL5A1 |
| | CXCL6 | CD274 | DCN | CXCL8 | CD27 | COL5A2 |
| | CXCL8 | CD3D | EFEMP1 | FCGR3B | CD37 | COL8A1 |
| | DMBT1 | CD52 | FBLN1 | FPR1 | CD38 | COL8A2 |
| | FCGR3B | CD8A | FNDC1 | G0S2 | CD3D | COLEC12 |
| | FPR1 | CXCL10 | GAS1 | HCAR3 | CD48 | COMP |
| | G0S2 | CXCL13 | GREM1 | IL1B | CD52 | DCN |
| | HCAR3 | CXCL9 | MAB21L2 | IL1RN | CD8A | DPYSL3 |
| | IL1B | GBP1 | MGP | IL24 | CXCL13 | EPYC |
| | IL1RN | GBP4 | MSRB3 | IL6 | CXCL9 | FBLN1 |
| | IL6 | GBP5 | MYL9 | MCEMP1 | GATA3 | FNDC1 |
| | LYZ | GIMAP6 | PCDH7 | MMP1 | GBP4 | GAS1 |
| | MMP1 | GNLY | PLN | MMP3 | GBP5 | GLT8D2 |
| | MMP3 | GPR171 | POSTN | PROK2 | GNLY | GXYLT2 |
| | PLEK | GZMA | SFRP2 | S100A12 | GPR171 | HOPX |
| | PPBP | GZMH | SFRP4 | S100A8 | GZMA | LOX |
| | PROK2 | GZMK | SPOCK1 | S100A9 | GZMB | LOXL1 |
| | PTGS2 | HLA-DMA | SULF1 | TFPI2 | GZMH | MFAP5 |
| | REG1A | HLA-DMB | SYNPO2 | TREM1 | GZMK | MXRA5 |
| | REG1B | HLA-DPB1 | TAGLN | | HLA-DMA | POSTN |
| | REG3A | HLA-DRB1 | THBS2 | | HLA-DOA | RGS4 |
| | S100A12 | IDO1 | TNS1 | | IDO1 | SFRP2 |
| | S100A8 | IKZF1 | VCAN | | IKZF1 | SFRP4 |
| | S100A9 | IL18BP | | | IL10RA | SPOCK1 |
| | SRGN | IRF1 | | | IL2RB | SPON1 |
| | TNFAIP6 | ITGAL | | | IRF1 | TWIST1 |
| | TREM1 | ITK | | | ITGAL | VCAN |
| | | NCKAP1L | | | ITK | VGLL3 |
| | | NKG7 | | | LAMP3 | ZFPM2 |
| | | PRF1 | | | NKG7 | |
| | | PSMB9 | | | NLRC3 | |
| | | RARRES3 | | | PSMB9 | |
| | | RASGRP1 | | | RASGRP1 | |
| | | SLAMF7 | | | SLAMF7 | |
| | | STAT1 | | | STAT1 | |
| | | TAP1 | | | TAGAP | |
| | | TRAC | | | TRAC | |
| | | TRAF3IP3 | | | TRAF3IP3 | |
| | | TRBC1 | | | TRBC1 | |
| | | UBE2L6 | | | | |
| | | WARS | | | | |

## Appendix table S3

**FACS**

| Antigen | Clone | Reactivity | Supplier | Cat. number | Lot number | Dilution |
|---|---|---|---|---|---|---|
| CD11b | M1/70 | Human, mouse | Biolegend | 101241 | B235643 | 1/400 |
| CD14 | HCD14 | Human | Biolegend | 325603 | B211976 | 1/100 |
| CD15 | HI98 | Human | Biolegend | 301907 | 7068674 | 18264 |
| CD206 | C068C2 | Mouse | Biolegend | 141711 | B230155 | 1/50 |
| CD3 | HIT3a | Human | Biolegend | 300317 | B241860 | 1/100 |
| CD4 | GK1.5 | Mouse | in house | not applicable | not applicable | 1/800 |
| CD45 | HI30 | Human | Biolegend | 304041 | B242281 | 1/100 |
| CD45 | M1/89 | Mouse | in house | not applicable | not applicable | 1/400 |
| CD66b | G10F5 | Human | Biolegend | 305105 | B221033 | 1/200 |
| CD8 | YTS169.4 | Mouse | in house | not applicable | not applicable | 1/1600 |
| EpCAM | G8.8 | Mouse | Biolegend | 118207 | B199448 | 1/100 |
| FOXP3 | 236A/E7 | Human, Monkey | eBioscience | 14-4777-80 | E04269-1631 | 1/100 |
| Gr1 | RB6-8C5 | Mouse | in house | not applicable | not applicable | 1/800 |
| HLA-DR | L243 | Human | Biolegend | 307635 | B223586 | 1/50 |
| IFNg | XMG1.2 | Mouse | Tonbo | 20-7311-U02 | C711111716202 | 1/100 |
| Ly6C | HK1.4 | Mouse | Biolegend | 128013 | B236102 | 1/400 |
| Ly6G | 1A8 | Mouse | Biolegend | 127615 | B218783 | 1/100 |
| MHCII | M5/114.15.2 | Mouse | Biolegend | 107603 | B189443 | 1/800 |

**MACS**

| Antigen | Clone | Reactivity | Supplier | Cat. number | Lot number | Dilution |
|---|---|---|---|---|---|---|
| CD3 | HIT3a | Human | Biolegend | 300303 | B230221 | 1/100 |
| CD66b | G10F5 | Human | Biolegend | 305120 | B215969 | 1/100 |
| Ly6G | 1A8 | Mouse | Biolegend | B205345 | B205345 | 1/100 |

**IHC-Paraffin**

| Antigen | Clone | Reactivity | Supplier | Cat. number | Lot number | Dilution |
|---|---|---|---|---|---|---|
| CD3 | Sp7 | Human, Mouse, Ra | Abcam | ab16669 | | 1/100 |
| CD8 | C8/144B | Human | eBioscience | 14-0085 | E15903-103 | 1/200 |
| CD66b | G10F5 | Human | Biolegend | 305102 | B197492 | 1/600 |
| MMP9 | 5G3 | Human | Thermo | MA5-15886 | TI2443744 | 1/400 |
| S100A9 | Polyclonal | Human, Mouse, Ra | Novus | NB110-8972 | A-2 | 1/5000 |
| MMP9 | Polyclonal | Mouse | Abcam | ab38898 | Gift of D Hanahan (EPFL) | 1/400 |

**IHC-Frozen**

| Antigen | Clone | Reactivity | Supplier | Cat. number | Lot number | Dilution |
|---|---|---|---|---|---|---|
| CD45 | 30-F11 | Mouse | BD Bioscien | 5505539 | | 1/200 |
| Gr1 | RB6-8C5 | Mouse | in house | not applicable | not applicable | 1/400 |

***In vivo* neutralisation**

| Antigen | Clone | Reactivity | Supplier | Cat. number | Lot number | Dilution |
|---|---|---|---|---|---|---|
| CD4 | YTS191.1 | Mouse | in house | not applicable | not applicable | 10 mg/kg body weight |
| CD8 | YTS169.4 | Mouse | in house | not applicable | not applicable | 10 mg/kg body weight |
| Gr1 | RB6-8C5 | Mouse | in house | not applicable | not applicable | 20 mg/kg body weight |

***In vitro* neutralisation**

| Antigen | Clone | Reactivity | Supplier | Cat. number | Lot number | Dilution |
|---|---|---|---|---|---|---|
| CD73 | TY/23 | Mouse | BioXcell | BE0209 | 616317J1 | 2 µg/ml |
| IL10 | JES5-2A5 | Mouse | Biolegend | 504903 | B178143 | 2 µg/ml |
| Tgfb1 | 11D1 | Mouse | BioXcell | BE0057 | 642917J1 | 2 µg/ml |

**Appendix table S4**

| Figure | Panel | exact p-value |
|---|---|---|
| 1 | B | Blood TCRbeta+ cells: p=0.00000006; Tumor TCRb+ cells: p=0.0155 |
| 1 | C | Tumor burden: p=0.0272; Tumor number: p=0.0174 |
| 2 | A | Colon vs Tumor: p=0.0000009 |
| 2 | C | Colon vs Tumor: p=0.00007727 |
| 2 | E | Colon vs Tumor: p=0.00002965 |
| 2 | F | Colon vs Tumor: p=0.00042612 |
| 2 | H | Colon vs Tumor: p=0.0005 |
| 2 | I | Colon vs Tumor: p=0.0303 |
| 2 | K | Colon vs Tumor: p=0.0367 |
| 3 | A | T cells + neutrophils vs T only 1:5: p=0.0434 |
| 3 | A | T cells + neutrophils vs T only 1:2: p=0.0011 |
| 3 | A | T cells + neutrophils vs T only 1:1: p=0.0026 |
| 3 | A | T cells + macrophages vs T only 1:1: p=0.0145 |
| 3 | C | Neutrophils IgG+DMSO vs aGr1+CXCR21: p=0.0167 |
| 3 | D | Tumor size IgG+DMSO vs aGr1+CXCR21: p=0.0075 |
| 3 | F | Tumor Tregs IgG+DMSO vs aGr1+CXCR21: p=0.0073 |
| 3 | G | Tumor activated T cells IgG+DMSO vs aGr1+CXCR21: p=0.0040 |
| 3 | G | Tumor Th17 T cells IgG+DMSO vs aGr1+CXCR21: p=0.0014 |
| 4 | E | T+Ne+TGFBRi vs T+Ne: p=0.00024829 |
| 4 | F | T+Ne vs T only: p=0.0376; T+Ne+TGFBRi vs T+Ne: p=0.0349 |
| 4 | G | T+Ne+MMPi vs T+Ne: p=0.0038 |
| 4 | H | T+Ne+TGFBRi vs T+Ne: p=0.0011 |
| 4 | H | T+Ne+TGFBRi+MMPi vs T+Ne: p=0.0009 |
| 4 | H | T+Ne+TGFBRi+MMPi vs T+Ne+TGFBRi: p=0.0033 |
| 5 | C | pSMAD3+ cells MMP2/9i vs Vehicle: p=0.0021. pSMAD3 intensity/cell MMP2/9i vs Vehicle: p=0.0013 |
| 5 | E | Tumor size TGFBRi vs Vehicle: p=0.0450. Tumor size MMP2/9i vs Vehicle: p=0.0280 |
| 6 | C | S100A9+ cells Tumor center vs tumor border: p=0.0078. S100A9+ cells Tumor border vs adjacent benign: p=0.0097. |
| 6 | C | CD8+ cells Tumor border vs adjacent benign: p=0.0261. S100A9+ cells Tumor center vs adjacent benign: p=0.0024. |
| 6 | E | TCGA cohort; neutrophil-high/TGFbeta-high vs Neutrophil-low/TGFbeta-low p=3.8e-8 |
| 6 | E | TCGA cohort; neutrophil-high/TGFbeta-high vs Neutrophil-high/TGFbeta-low p=1.9e-5 |
| 6 | E | TCGA cohort; neutrophil-low/TGFbeta-high vs Neutrophil-low/TGFbeta-low p=0.00024 |
| 6 | E | GSE39582 cohort; neutrophil-high/TGFbeta-high vs Neutrophil-low/TGFbeta-low p=2.3e-9 |
| 6 | E | GSE39582 cohort; neutrophil-high/TGFbeta-high vs Neutrophil-high/TGFbeta-low p=6.4e-9 |
| 6 | E | GSE39582 cohort; neutrophil-low/TGFbeta-high vs Neutrophil-low/TGFbeta-low p=0.00048 |
| 6 | F | T cells + neutrophils vs T only 1:8: p=0.02325821 |
| 6 | F | T cells + neutrophils vs T only 1:4: p=0.00954330 |
| 6 | F | T cells + neutrophils vs T only 1:2: p<0.00000001 |
| 6 | F | T cells + neutrophils vs T only 1:1: p<0.00000001 |
| 6 | F | T cells + neutrophils vs T only 2:1: p<0.00000001 |
| 6 | G | T+Ne+TGFBRi vs T+Ne: p=0.0204 |
| EV1 | A | Colon vs Tumor: p=0.0009 |
| EV1 | E | Activated T cells Colon vs Tumor: p=0.0023 |
| EV1 | E | CTLs Colon vs Tumor: p=0.0423 |
| EV2 | A | aT+aNe vs Ctrl: p=0.0018 |
| EV2 | B | aNe vs Ctrl: p=0.0119. aT+aNe vs Ctrl: p=0.0011 |
| EV2 | C | aNe vs Ctrl: p=0.0486. aT+aNe vs aNe: p=0.0021 |
| S1 | B | IgG vs aCD4/aCD8: p=0.0383 |
| S2 | C | Blood CD8+ T cells: p=0.000064. Tumor CD8+ T cells: p=0.0057 |
| S2 | D | Tumor number Ctrl vs aCD4/aCD8: p=0.0043 |
| S4 | A | Colon vs Tumor: p=0.0002 |
| S4 | C | Colon vs Tumor: p=0.0106 |
| S4 | E | Colon vs Tumor: p=0.0383 |
| S6 | A | Tumor neutrophils Ctrl vs aGr1+CXCR2i: p=0.0001 |
| S6 | B | Blood neutrophils Ctrl vs aGr1+CXCR2i: p=0.000058 |
| S6 | C | Tumor burden Ctrl vs aGr1+CXCR2i: p=0.0312 |
| S6 | E | CD11b+ MHCII+ CD11c+ cells Control vs CSF1Ri: p=0.0059. CD11b+ MHCII+ CD206+ cells Control vs CSF1Ri: p=0.0035. |
| S8 | C | Galunisertib vs Vehicle: p<0.0001. SB431542 vs Vehicle: <0.0001. aTgfb vs Vehicle: p=0.000502 |
| S9 | A | Tumor S100A9+ cells Ctrl vs aGr1+CXCR2i: p=0.0144 |
| S9 | B | Tumor MMP9+ cells Ctrl vs aGr1+CXCR2i: p=0.0013 |
| S9 | C | pSMAD3 expression Ctrl vs aGr1+CXCR2i: p=0.0417. IGFBP7 expression Ctrl vs aGr1+CXCR2i: p=0.0109 |
| S11 | A | TCGA cohort; neutrophil-high/TGFbeta-high vs Neutrophil-low/TGFbeta-low p=1.1e-15 |
| S11 | A | TCGA cohort; neutrophil-high/TGFbeta-high vs Neutrophil-high/TGFbeta-low p=2.2e-9 |
| S11 | A | TCGA cohort; neutrophil-low/TGFbeta-high vs Neutrophil-low/TGFbeta-low p=7.7e-7 |
| S11 | A | GSE39582 cohort; neutrophil-high/TGFbeta-high vs Neutrophil-low/TGFbeta-low p=2.2e-16 |
| S11 | A | GSE39582 cohort; neutrophil-high/TGFbeta-high vs Neutrophil-high/TGFbeta-low p=0.0074 |
| S11 | A | GSE39582 cohort; neutrophil-low/TGFbeta-high vs Neutrophil-low/TGFbeta-low p=0.17 |
