# Supplementary material for: Neutrophils suppress tumor‐infiltrating T cells in colon cancer via matrix metalloproteinase‐mediated activation of TGFβ
Source: EMBO Mol Med. 2019 Dec 2;12(1):e10681. doi: 10.15252/emmm.201910681 (PMC6949488; doi:10.15252/emmm.201910681)
Supplement: Supplementary file 2 — Expanded View Figures PDF [file EMMM-12-e10681-s002.pdf]

Expanded View Figures

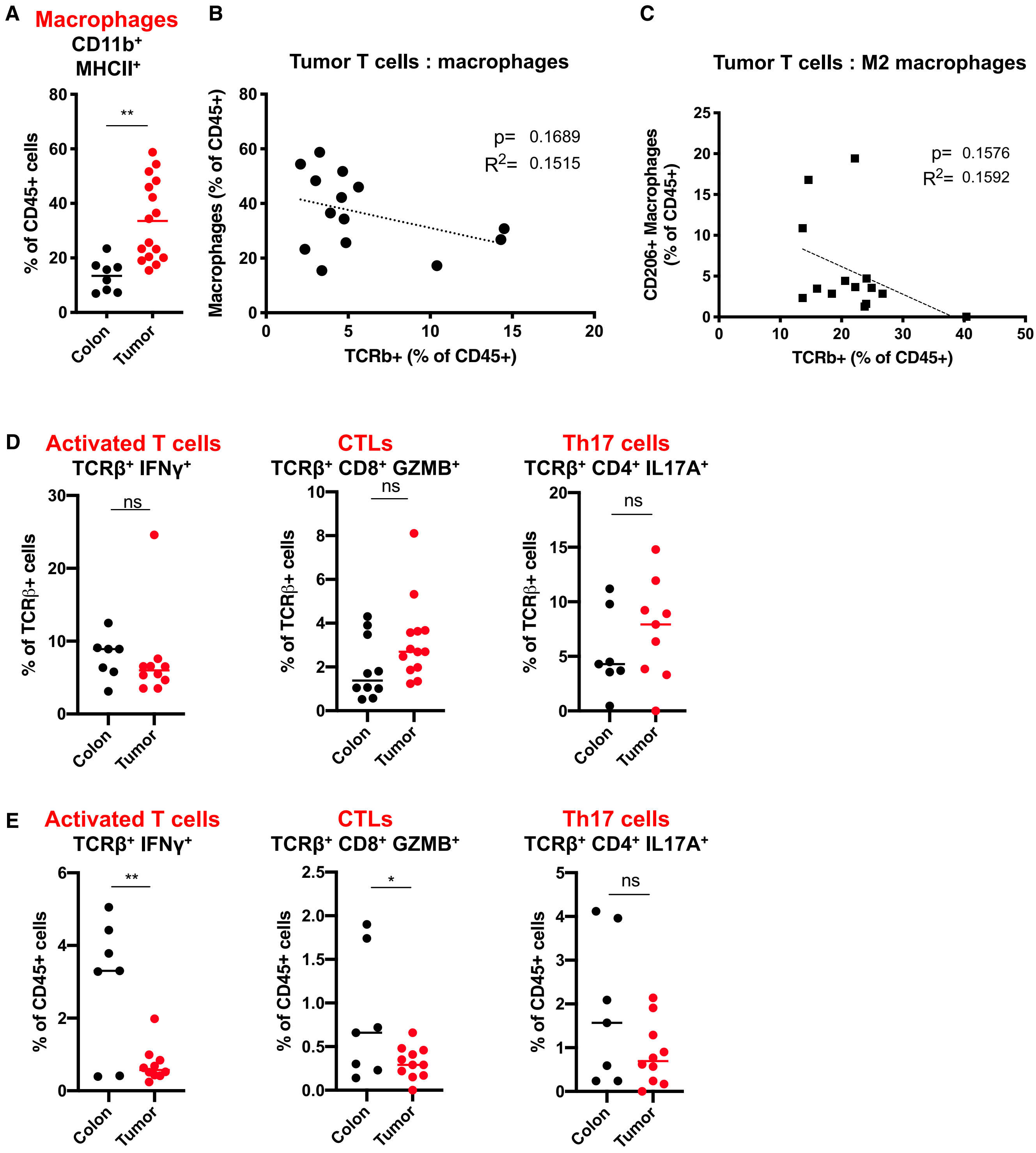

Figure EV1.

Figure EV1. Infiltration of macrophages and T-cell subsets in mouse colon tumors.

A Relative CD11b+ MHCII+ macrophage content in colon (n = 8) and tumors (n = 16).
B Correlation of relative CD11b+ MHCII+ macrophage content to sample-matched relative TCRβ+ T-cell content in colon tumors (n = 14).
C Correlation of CD206+ macrophage content to sample-matched T-cell content isolated from colon tumors (n = 14).
D, E IFNγ+, CD8+ GZMB+, and IL17A+ CD4+ effector T-cell subsets in normal colon and colon tumors displayed as percentage of total TCRβ+ T cells (D) or total CD45+ hematopoietic cells (E).

Data information: In (A–E), each dot represents an individual mouse. (D, left panel); Colon: n = 7. Tumor: n = 10. (D, middle panel); Colon: n = 10. Tumor: n = 13. (D, right panel); Colon: n = 7. Tumor: n = 9. (E, left panel); Colon: n = 7. Tumor: n = 10. (E, middle panel); Colon: n = 7. Tumor: n = 11. (E, right panel); Colon: n = 7. Tumor: n = 10. (A), (D), and (E), statistical analysis was performed by unpaired two-tailed Student's t-tests. *P < 0.05; **P < 0.01. Exact P-values are provided in Appendix Table S4. (B) and (C), statistical analysis was performed by linear regression.

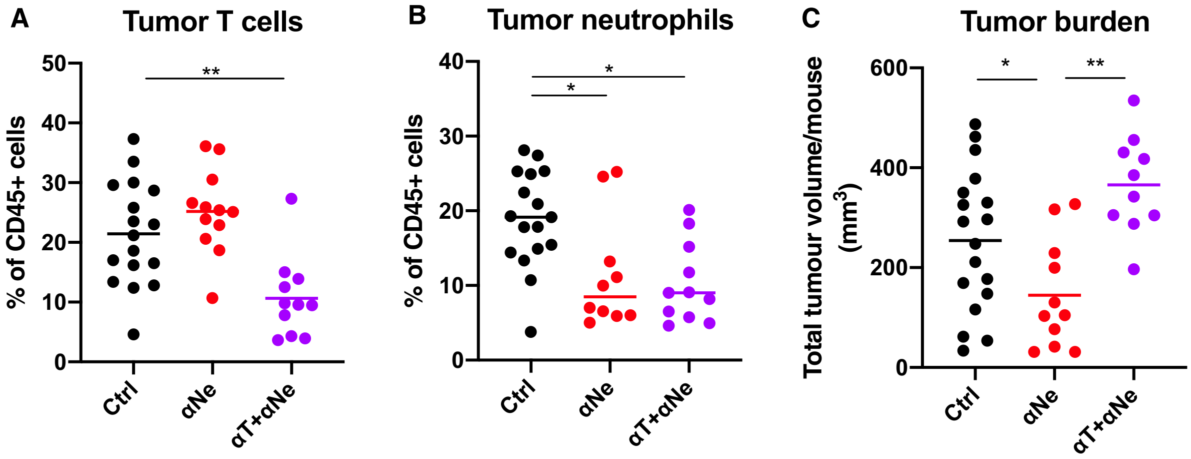

Figure EV2. Effect of neutrophil plus T-cell co-depletion on mouse colon tumor formation.

A–C *Apc*$^{fl/fl\text{-}Cdx2CreERT2}$ mice were treated with Tamoxifen and 1 day post-treatment injected with either IgG control, neutrophil depletion regimen (αGr1+CXCR2i; αNe) or with neutrophil depletion regimen plus T-cell depletion regimen (αCD4+αCD8+) (αT + αNe) for 5 weeks (A). Colons were then excised and analyzed by FACS for their content of CD45+ TCRβ+ T cells (A) and CD45+ CD11b+ Ly6C^{lo} SSC^{hi} neutrophils (B) and scored on the number and size of tumors (C).

Data information: In (A–C), each dot represents an individual mouse. (A) Ctrl: n = 17. αNe: n = 11. αT+αNe: n = 11. (B) Ctrl: n = 17. αNe: n = 10. αT+αNe: n = 11. (C) Ctrl: n = 18. αNe: n = 11. αT+αNe: n = 11. Statistical analysis was performed by unpaired two-tailed Student's t-tests. *P < 0.05; **P < 0.01. Exact P-values are provided in Appendix Table S4.

Figure EV3. TGFβ-signaling activation and neutrophil infiltration at different stages of adenoma formation.

A–C Co-immunostaining of the TGFβ signaling component pSMAD3 (brown) and the neutrophil marker S100A9 (gray-blue) on sections of *Apc*$^{fl/fl\text{-}Cdx2CreERT2}$ mouse colon adenomas representative of different stages of adenoma formation. (A and B) Sections of established adenoma and adjacent benign mucosa 14 weeks after tumor initiation. (A) Right panels are higher magnifications of indicated areas in left panel. Adenoma tissue (left panel, below) presents with strong pSMAD3 staining in adenoma, but weak pSMAD3 staining in the adjacent benign tissue (left panel, above). (B) High magnification of tumor tissue demonstrating pSMAD3 staining in epithelial cells (delineated by dashed black lines), as well as in tumor stromal cells, including endothelium (*) lining cells. (C) Section of an early, aberrant crypt focus-like colon lesion 3 weeks after tumor initiation.
D Immunostaining of the TGFβ target gene IGFBP7 in an early colon lesion 3 weeks after tumor initiation. Lower panels are higher magnifications of areas indicated in upper panel. IGFBP7 staining was mainly found in the stroma of the adenomatous lesion (left area), but not in adjacent benign mucosa (right panel).

Source data are available online for this figure.

© 2019 The Authors

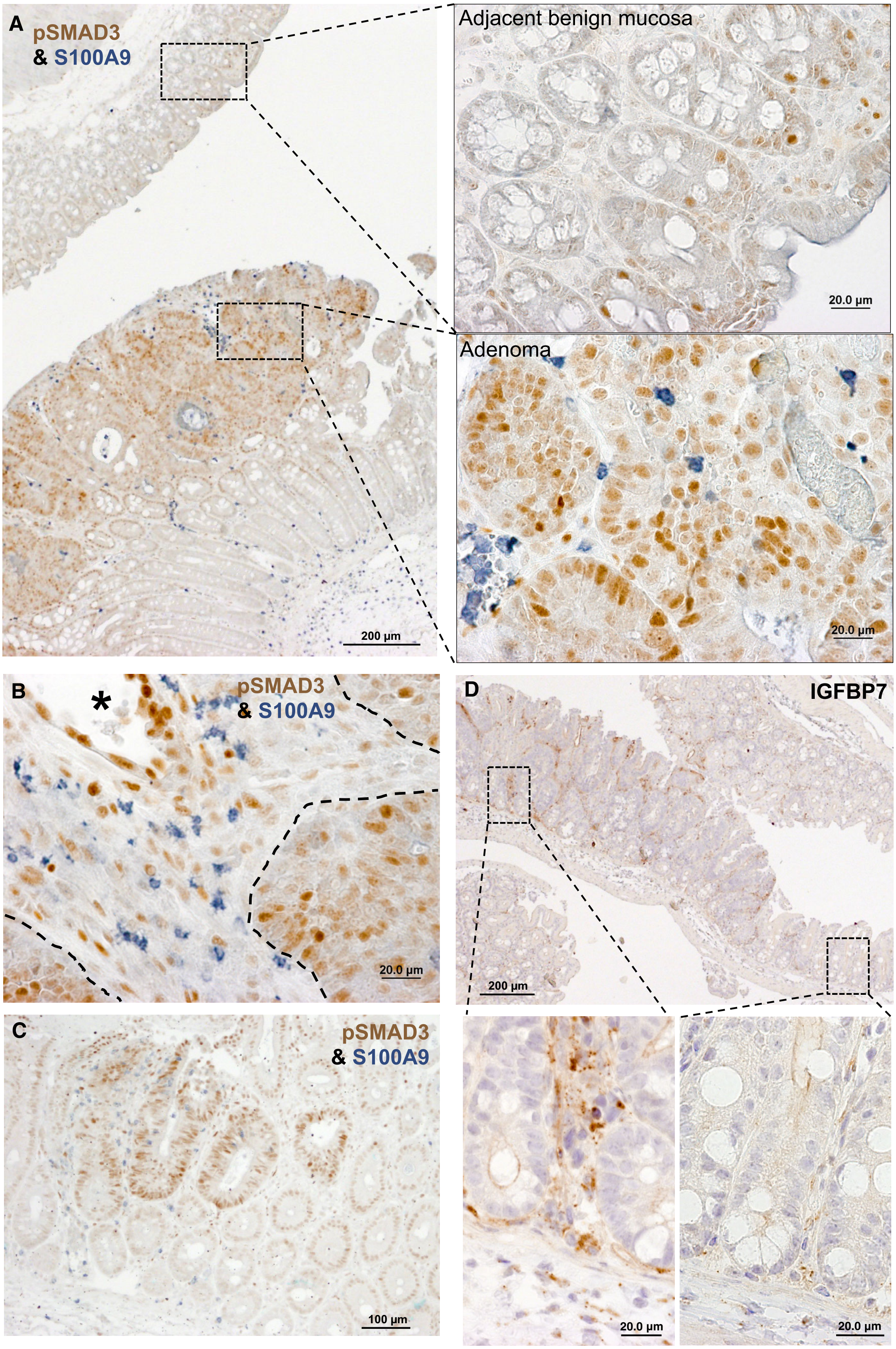
A
pSMAD3
& S100A9
200 µm
Adjacent benign mucosa
20.0 µm
Adenoma
20.0 µm
B
*
pSMAD3
& S100A9
20.0 µm
C
pSMAD3
& S100A9
100 µm
D
IGFBP7
200 µm
20.0 µm
20.0 µm

Figure EV3.
