# Supplementary figures and images for: Neutrophils suppress tumor‐infiltrating T cells in colon cancer via matrix metalloproteinase‐mediated activation of TGFβ
Source: EMBO Mol Med. 2019 Dec 2;12(1):e10681. doi: 10.15252/emmm.201910681 (PMC6949488; doi:10.15252/emmm.201910681)

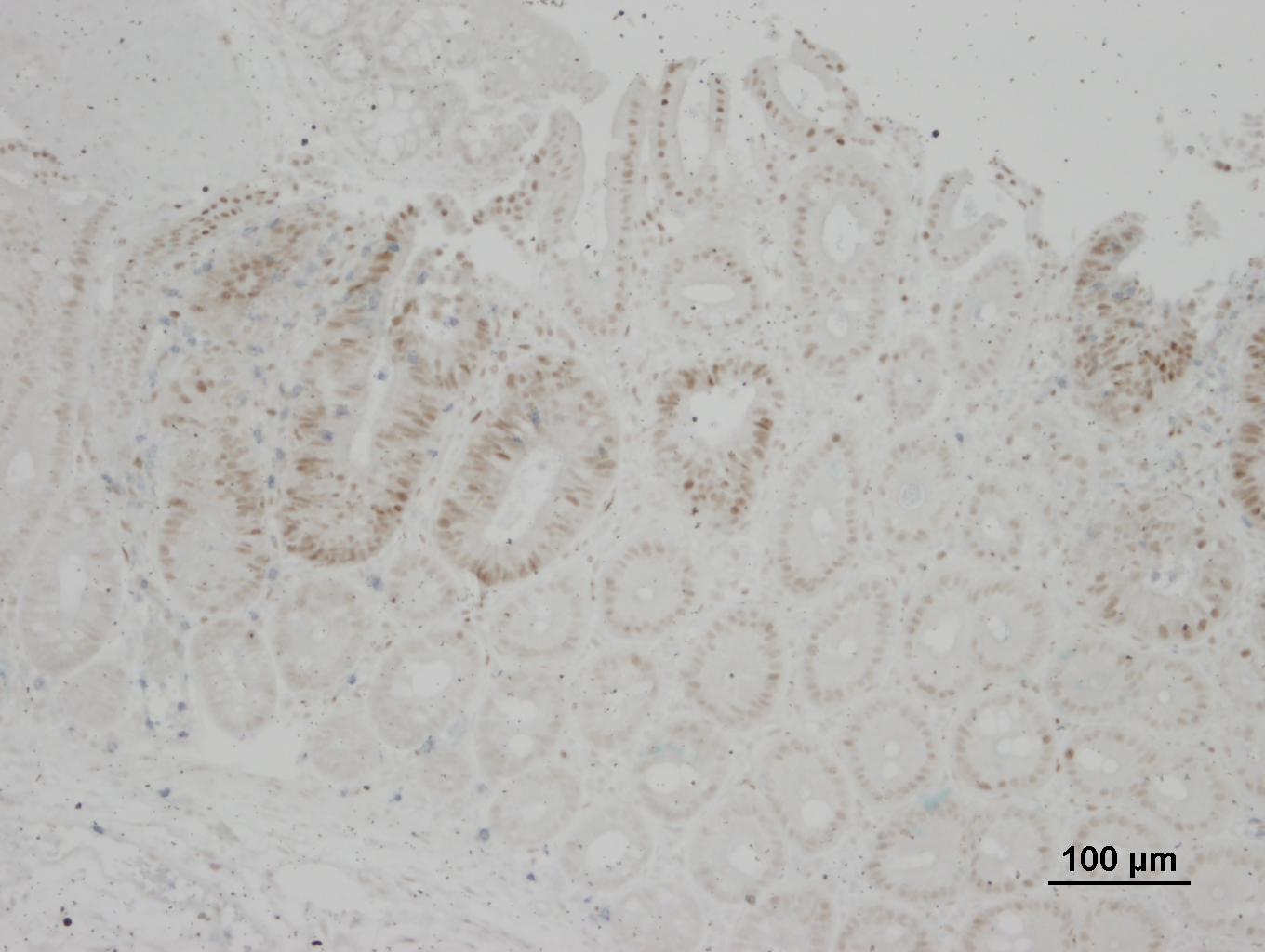

Supplement: Supplementary file 3 — Source Data for Expanded View [file EMMM-12-e10681-s008.zip › figEV3/FigEV3C.tiff]

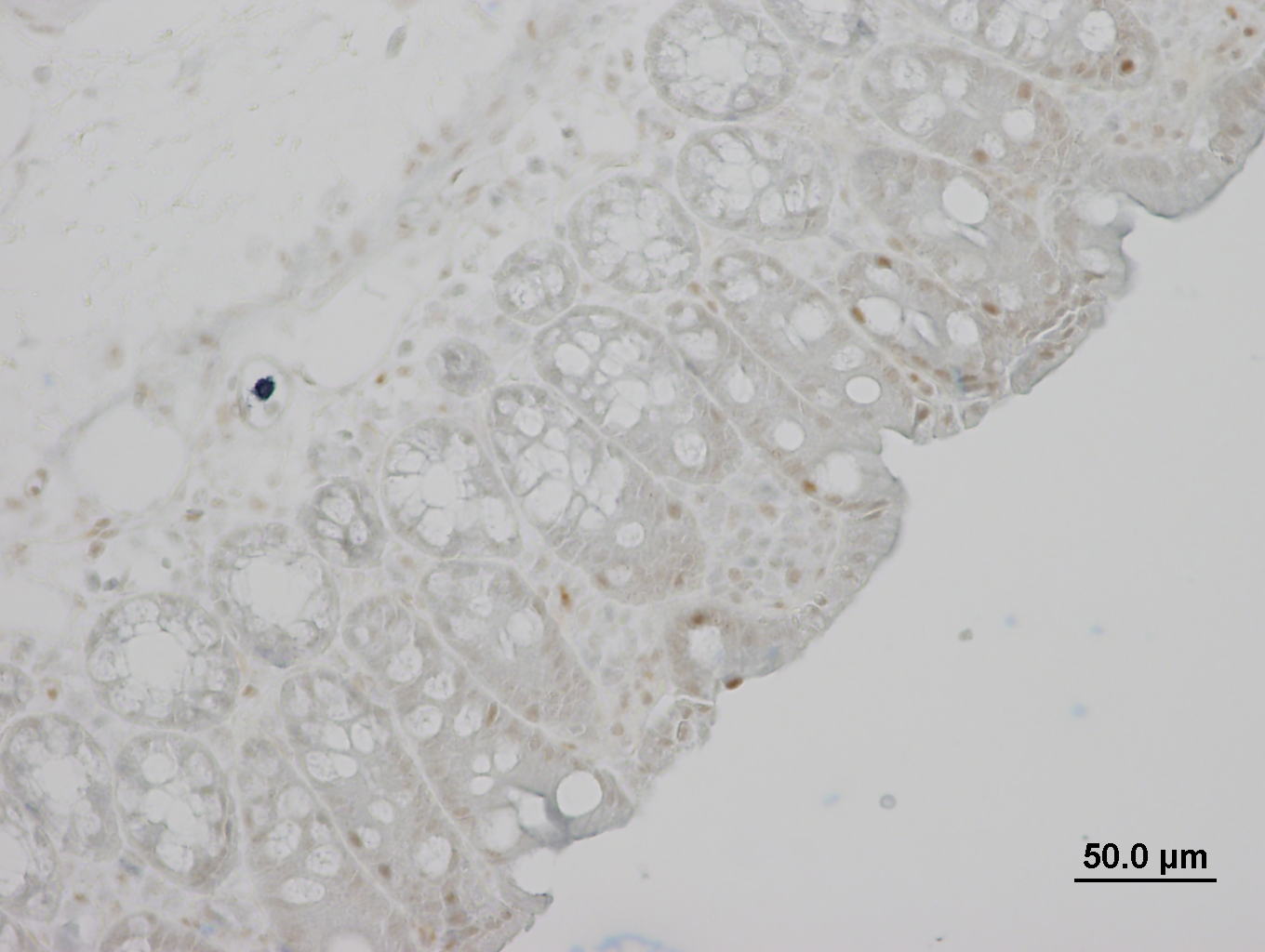

Supplement: Supplementary file 3 — Source Data for Expanded View [file EMMM-12-e10681-s008.zip › figEV3/FigEV3Aii.tiff]

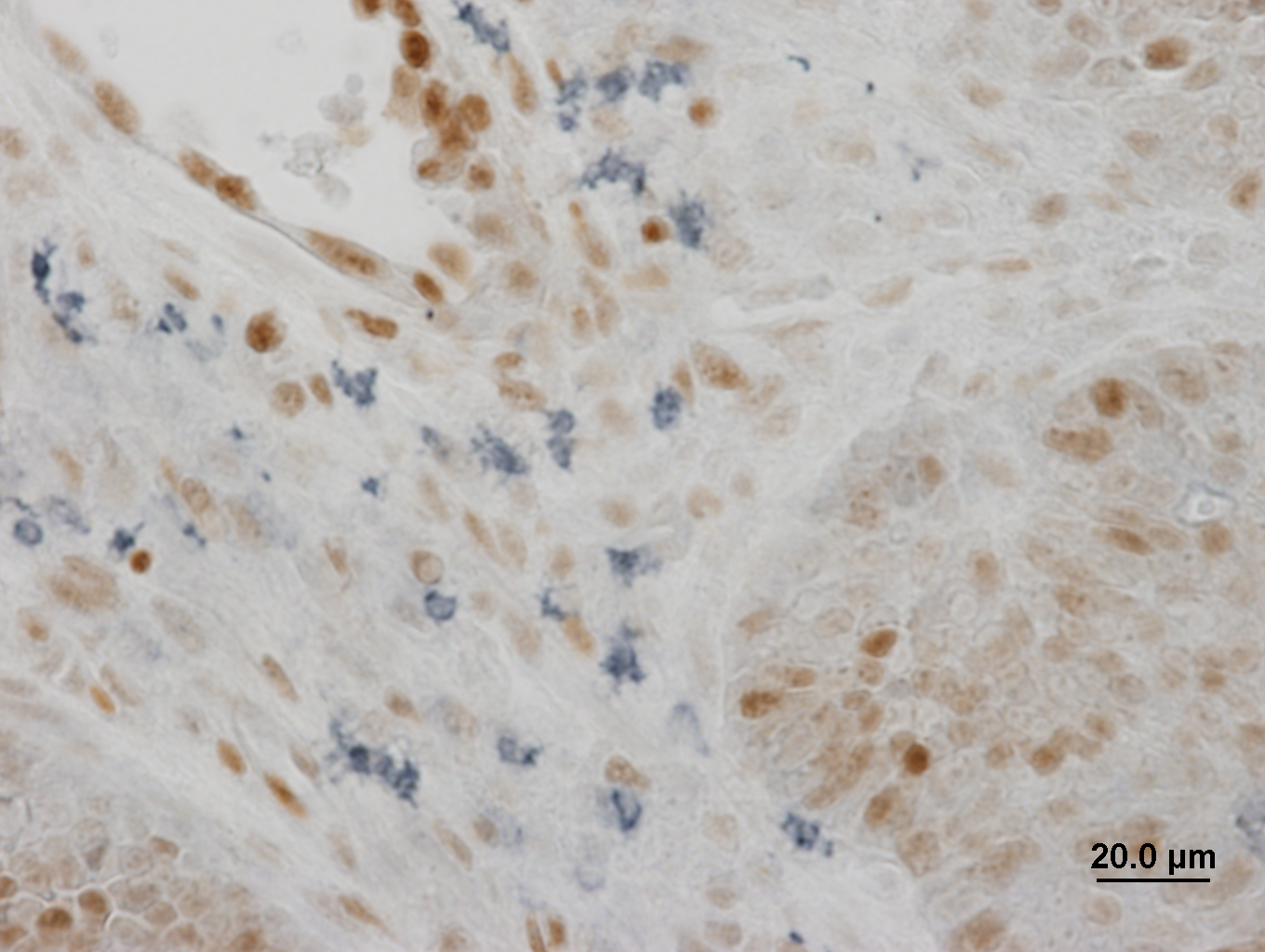

Supplement: Supplementary file 3 — Source Data for Expanded View [file EMMM-12-e10681-s008.zip › figEV3/FigEV3B.tiff]

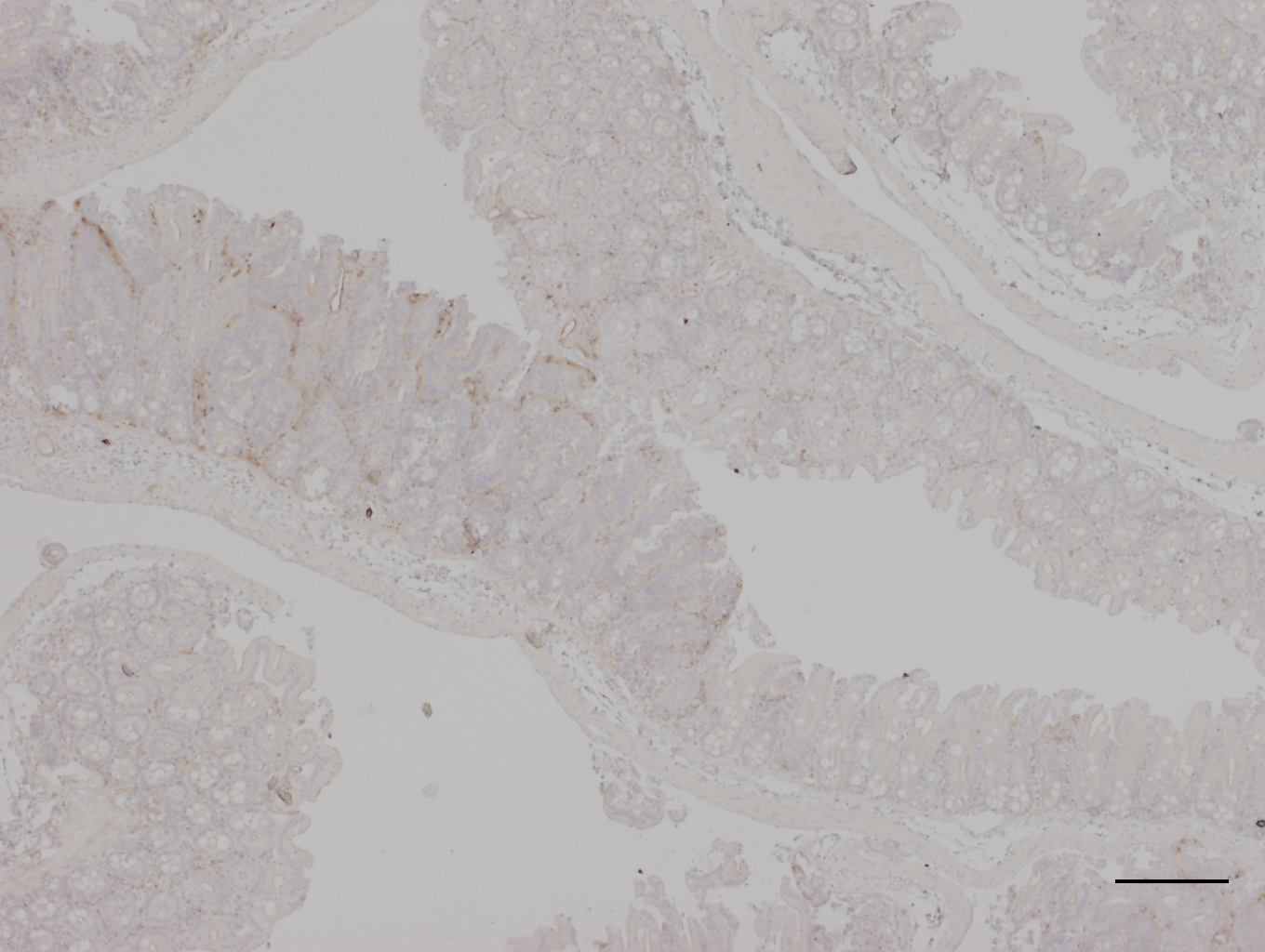

Supplement: Supplementary file 3 — Source Data for Expanded View [file EMMM-12-e10681-s008.zip › figEV3/FigEV3D.tiff]

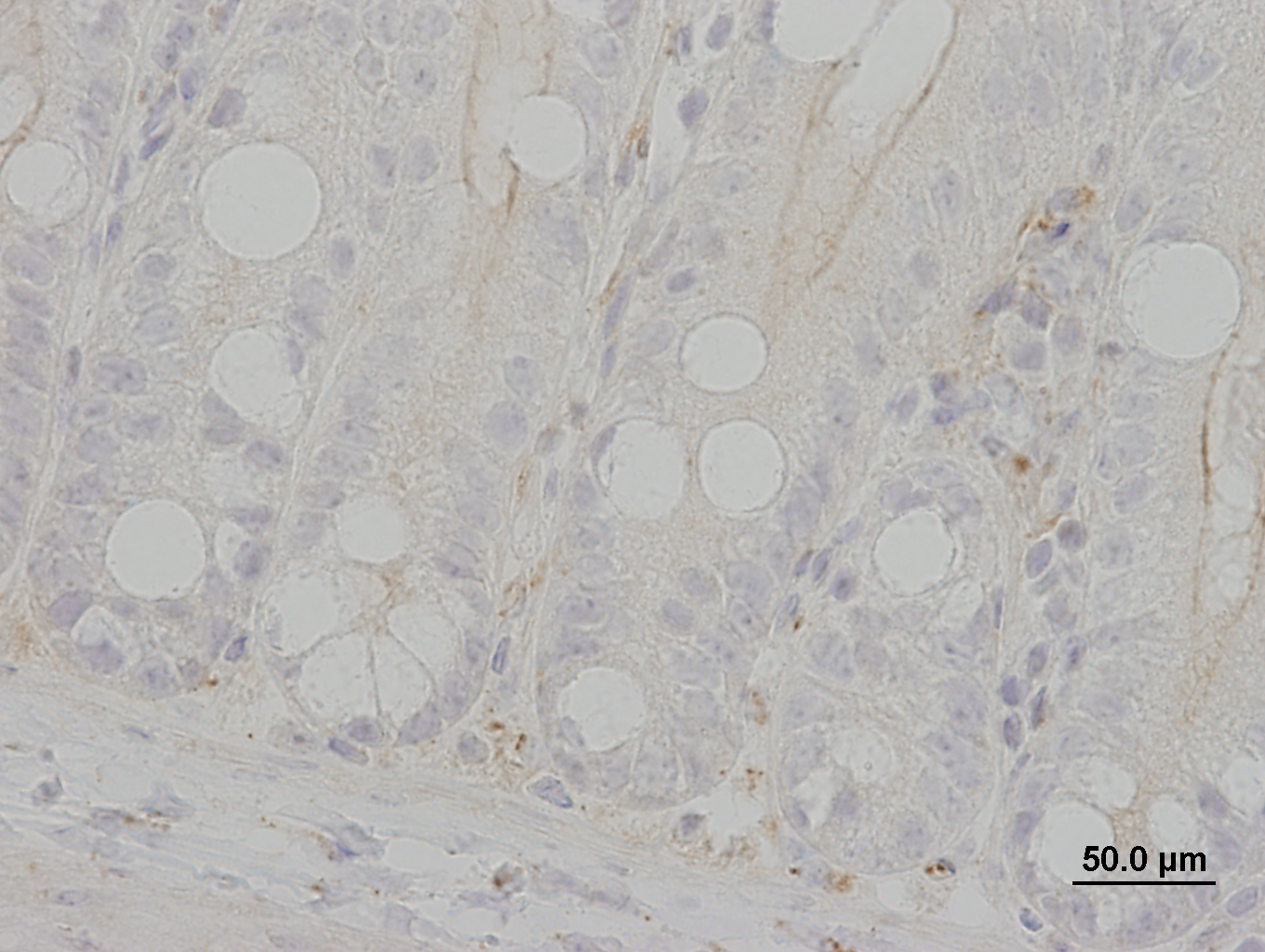

Supplement: Supplementary file 3 — Source Data for Expanded View [file EMMM-12-e10681-s008.zip › figEV3/FigEV3Diii.tiff]

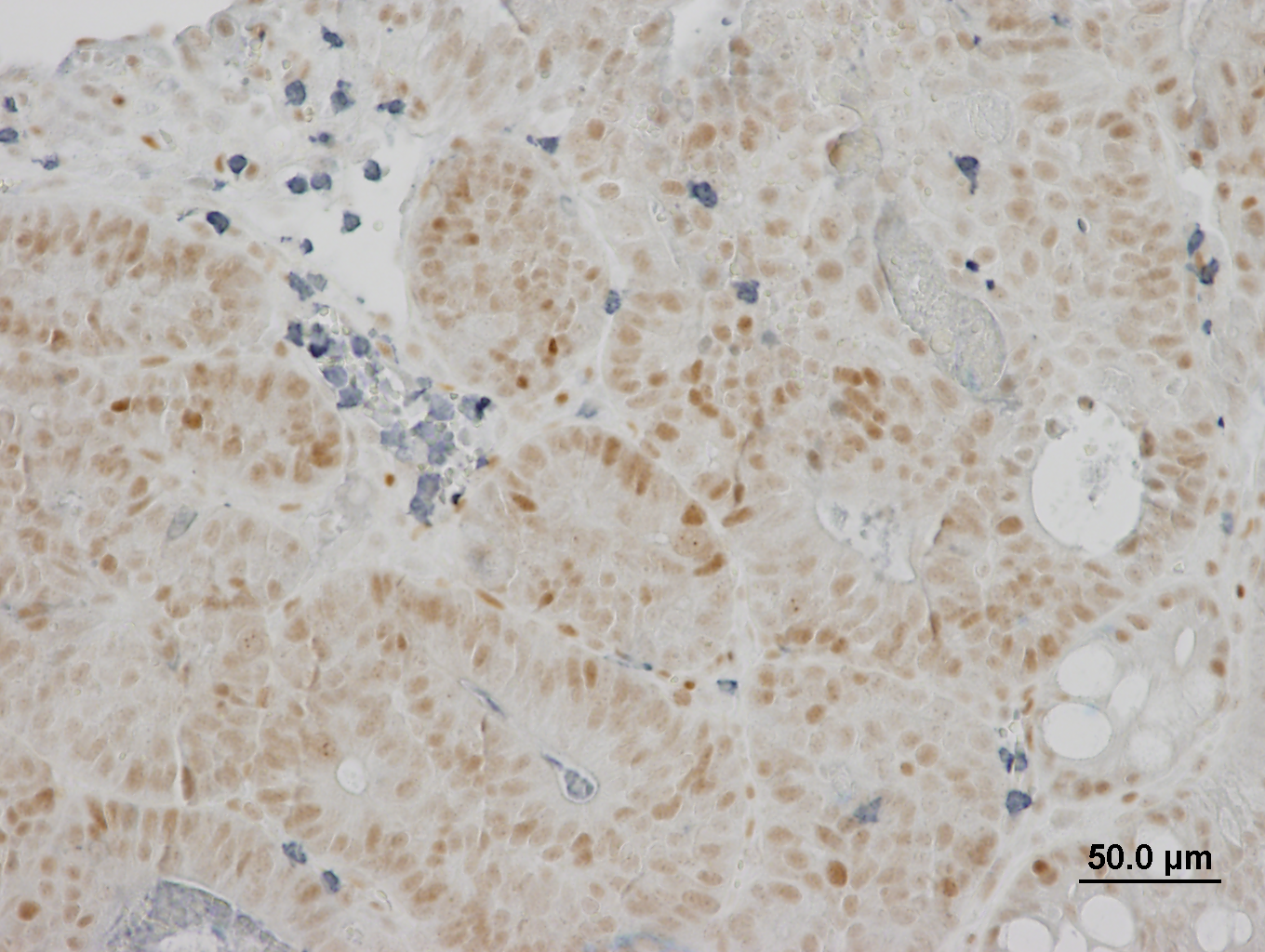

Supplement: Supplementary file 3 — Source Data for Expanded View [file EMMM-12-e10681-s008.zip › figEV3/FigEV3Aiii.tiff]

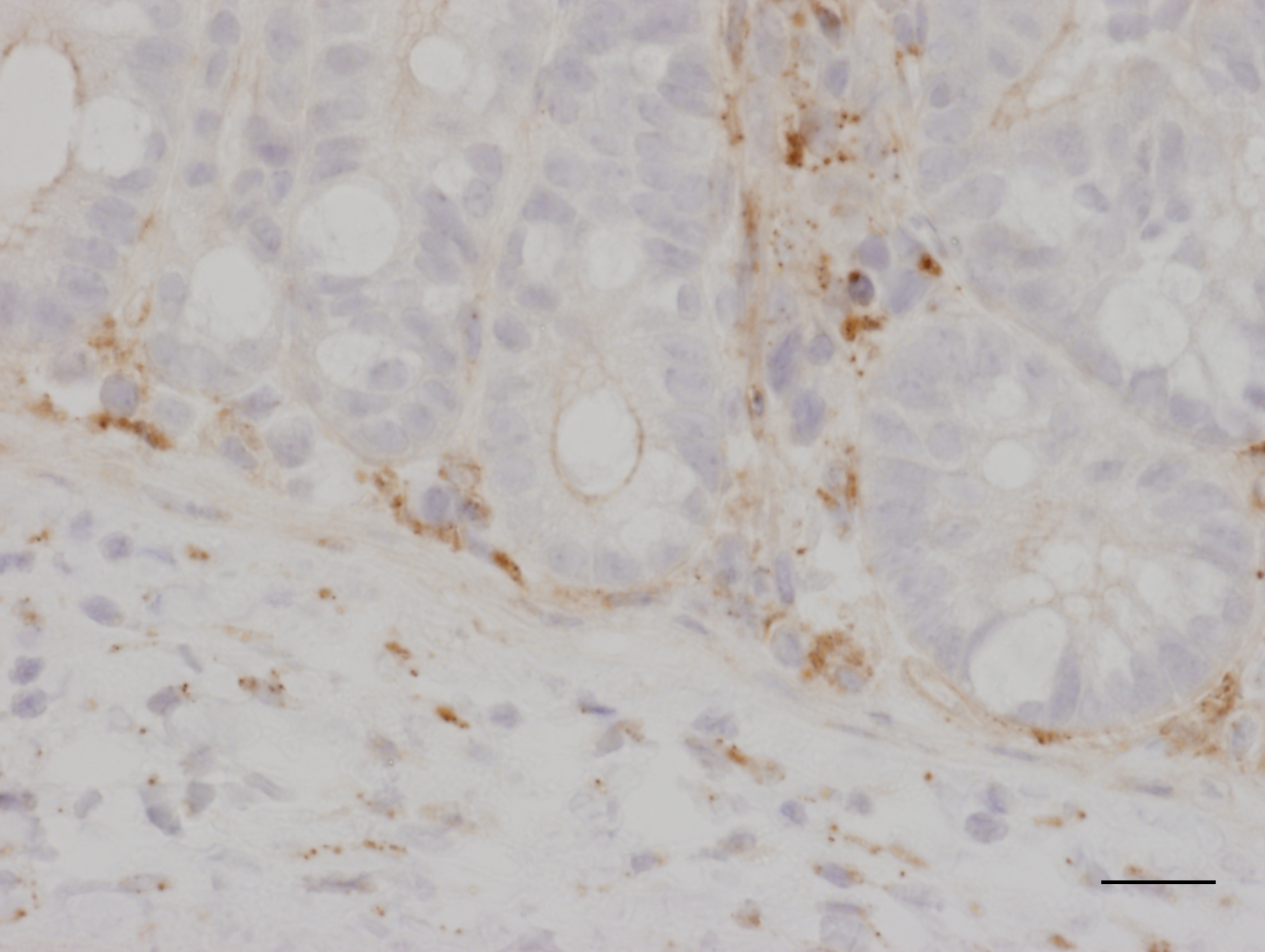

Supplement: Supplementary file 3 — Source Data for Expanded View [file EMMM-12-e10681-s008.zip › figEV3/FigEV3Dii.tiff]

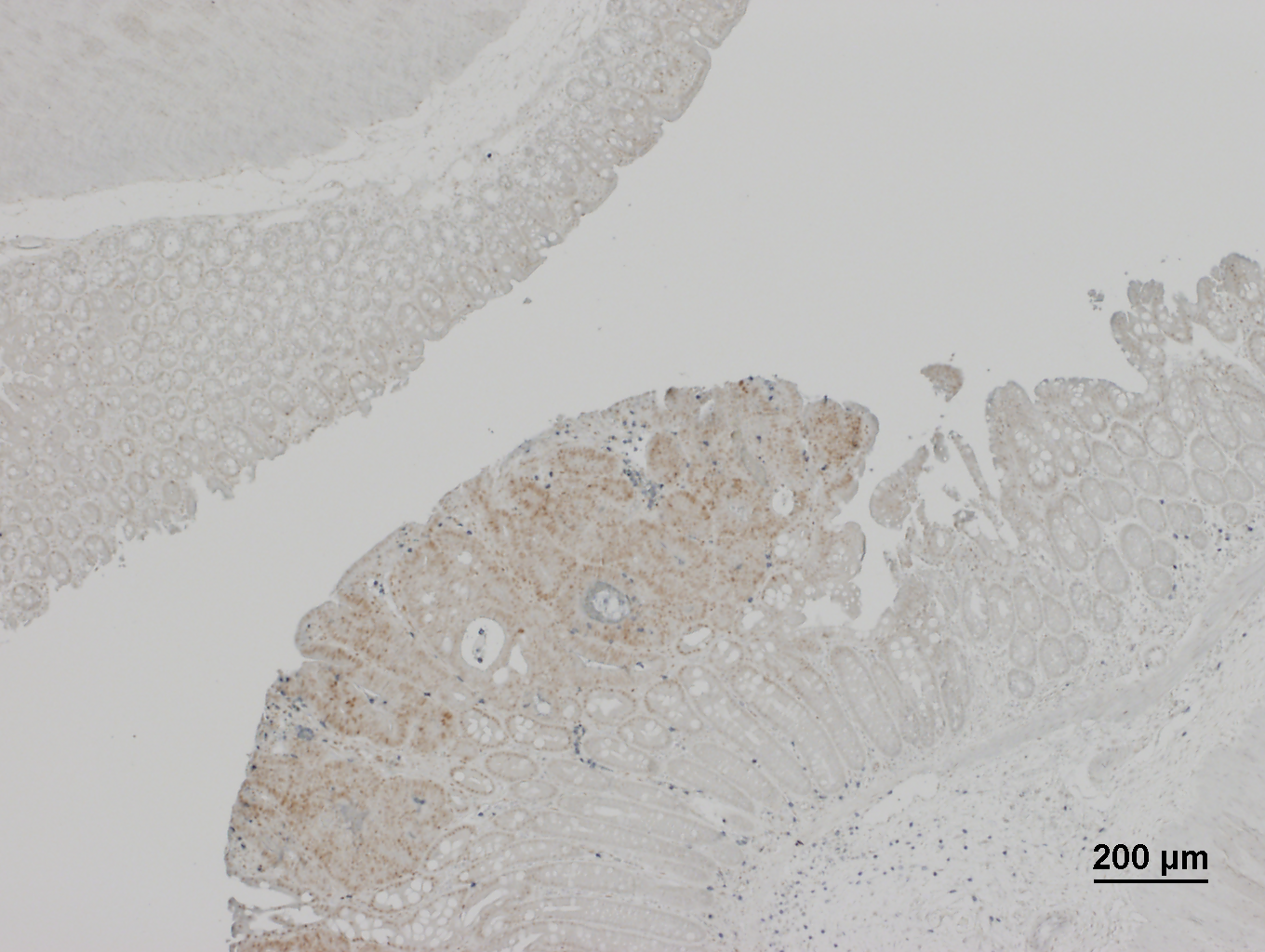

Supplement: Supplementary file 3 — Source Data for Expanded View [file EMMM-12-e10681-s008.zip › figEV3/FigEV3A.tiff]

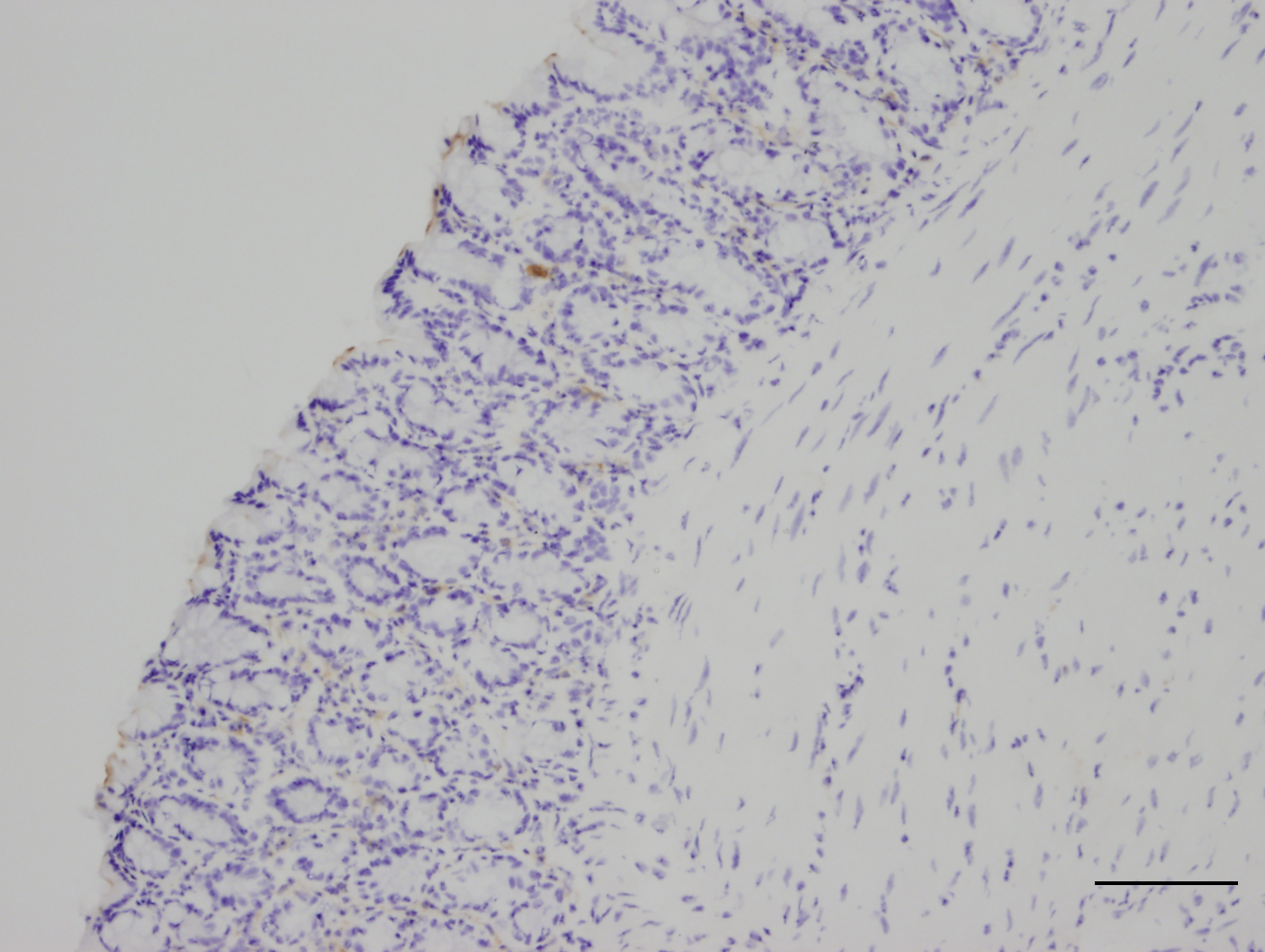

Supplement: Supplementary file 5 — Source Data for Figure 2 [file EMMM-12-e10681-s003.zip › fig2/Fig2Di.tiff]

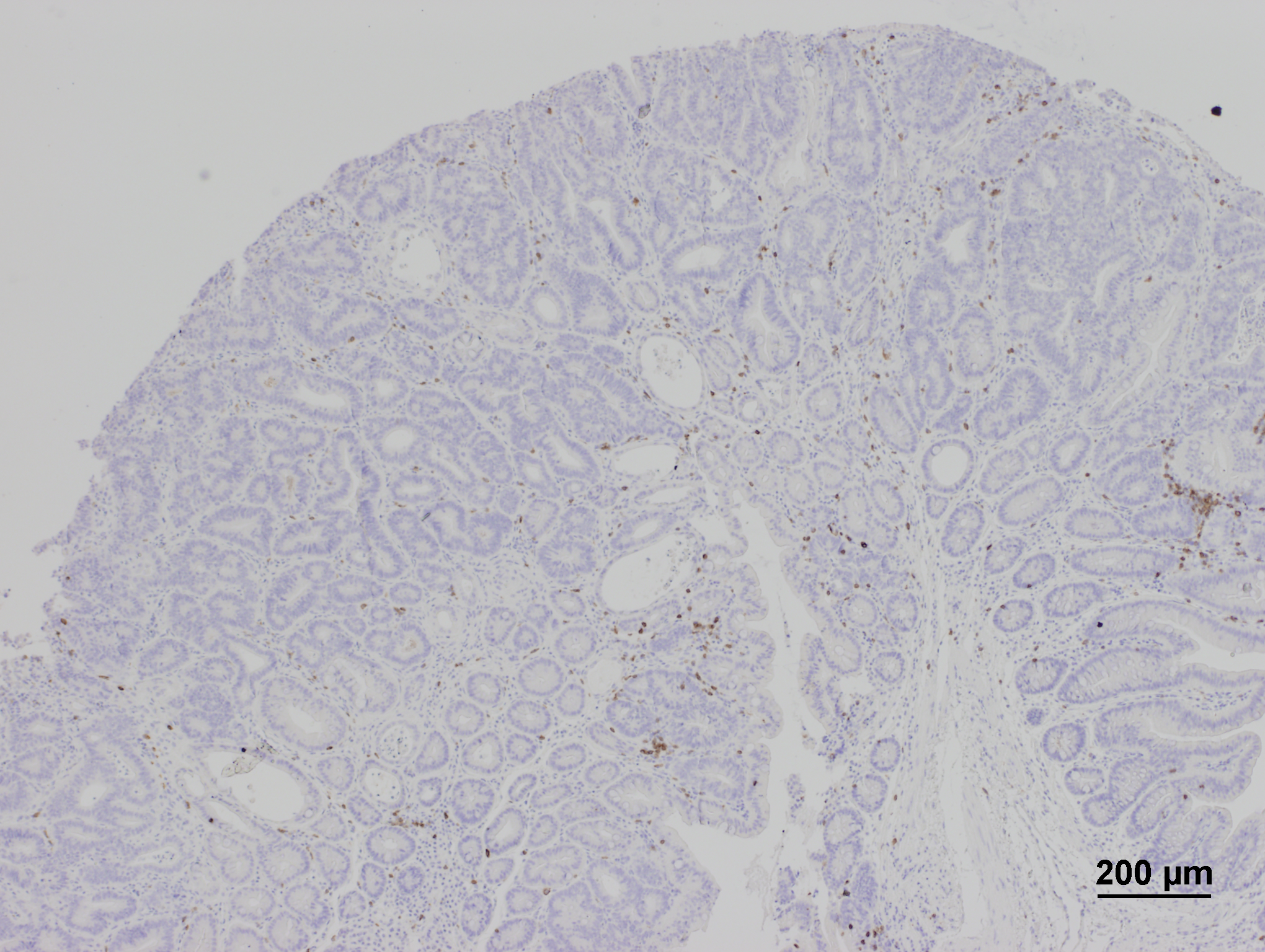

Supplement: Supplementary file 5 — Source Data for Figure 2 [file EMMM-12-e10681-s003.zip › fig2/Fig2Bii.tiff]

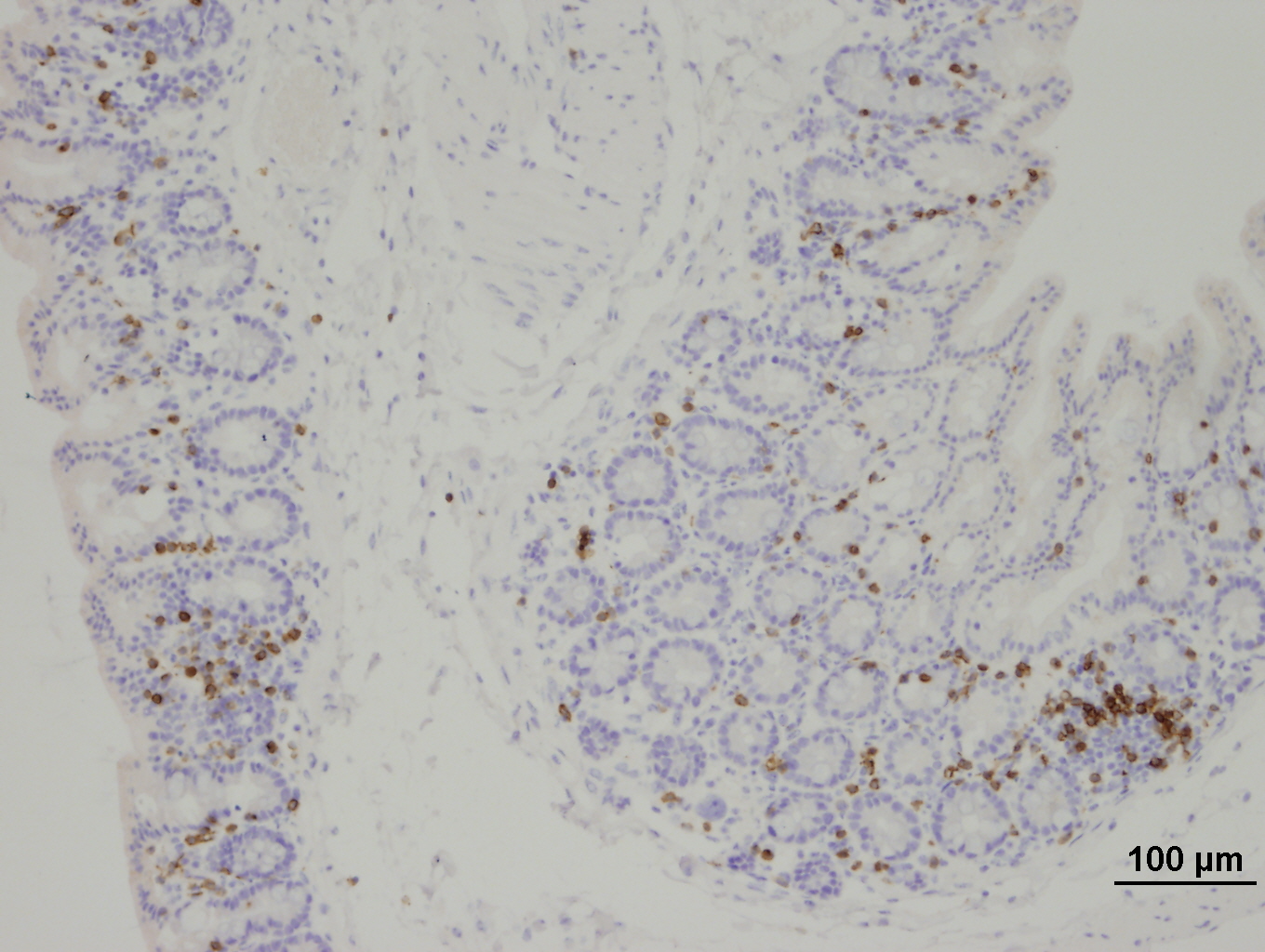

Supplement: Supplementary file 5 — Source Data for Figure 2 [file EMMM-12-e10681-s003.zip › fig2/Fig2Bi.jpg]

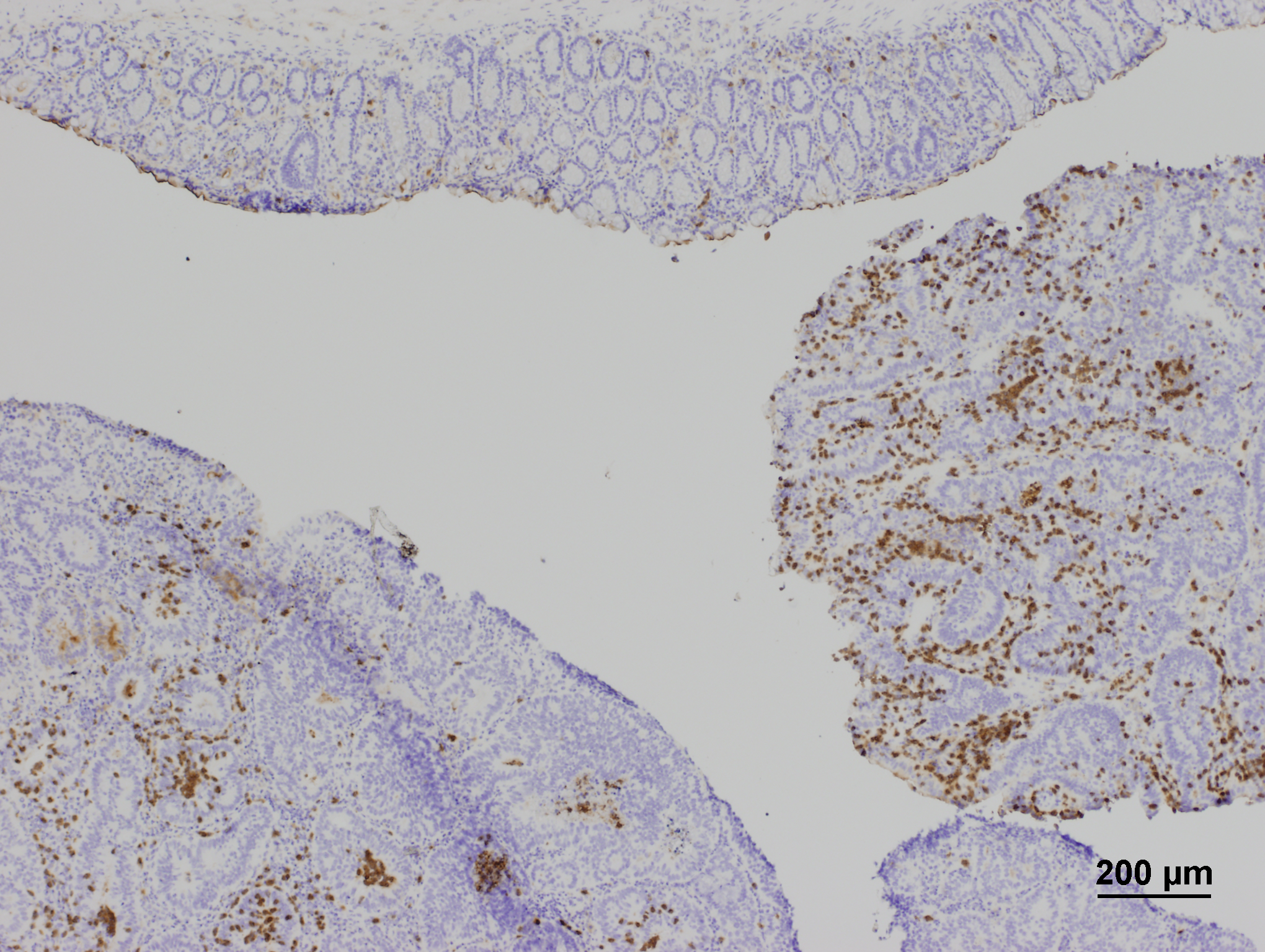

Supplement: Supplementary file 5 — Source Data for Figure 2 [file EMMM-12-e10681-s003.zip › fig2/Fig2Dii.tiff]

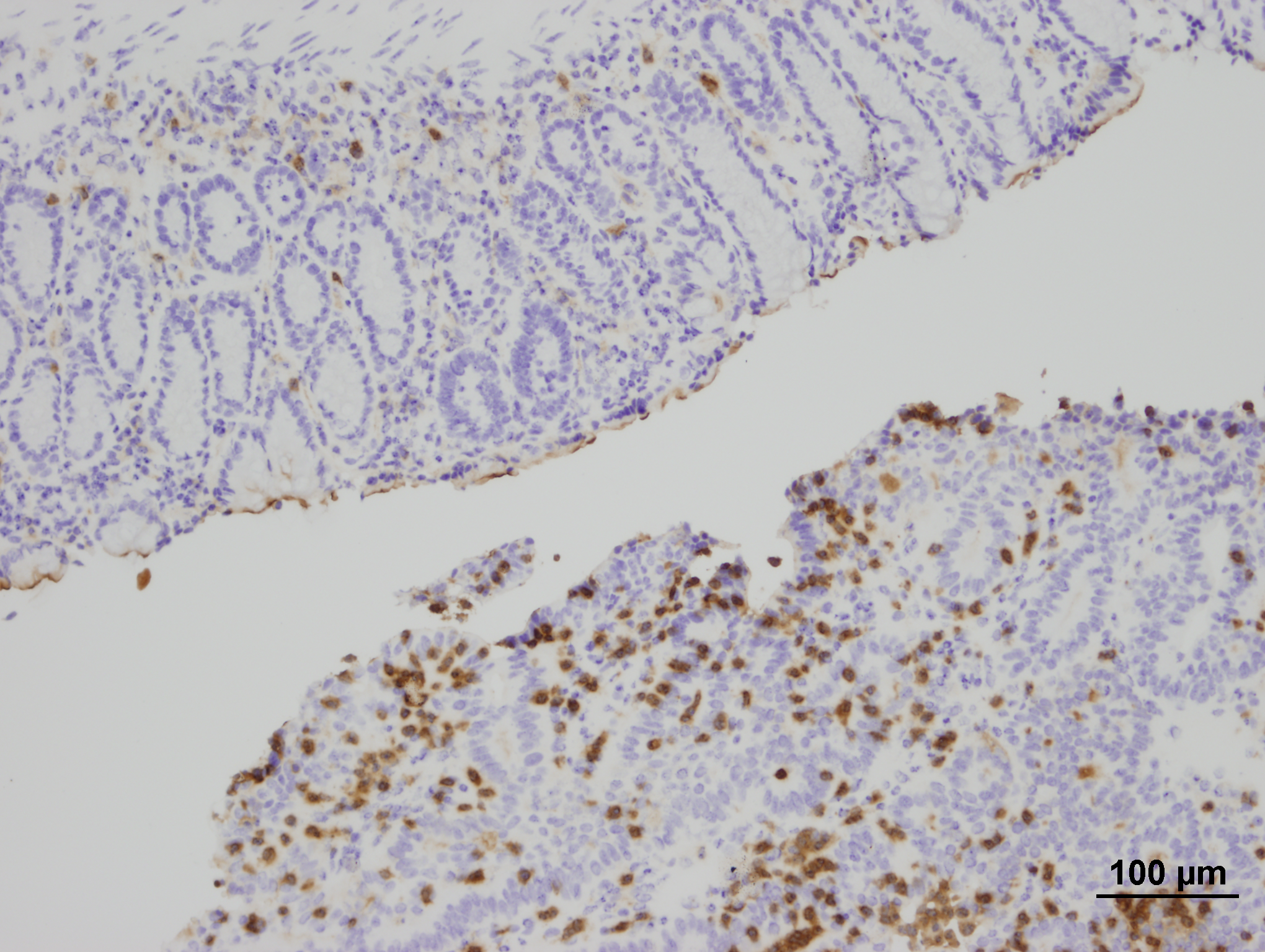

Supplement: Supplementary file 5 — Source Data for Figure 2 [file EMMM-12-e10681-s003.zip › fig2/Fig2Diii.tiff]

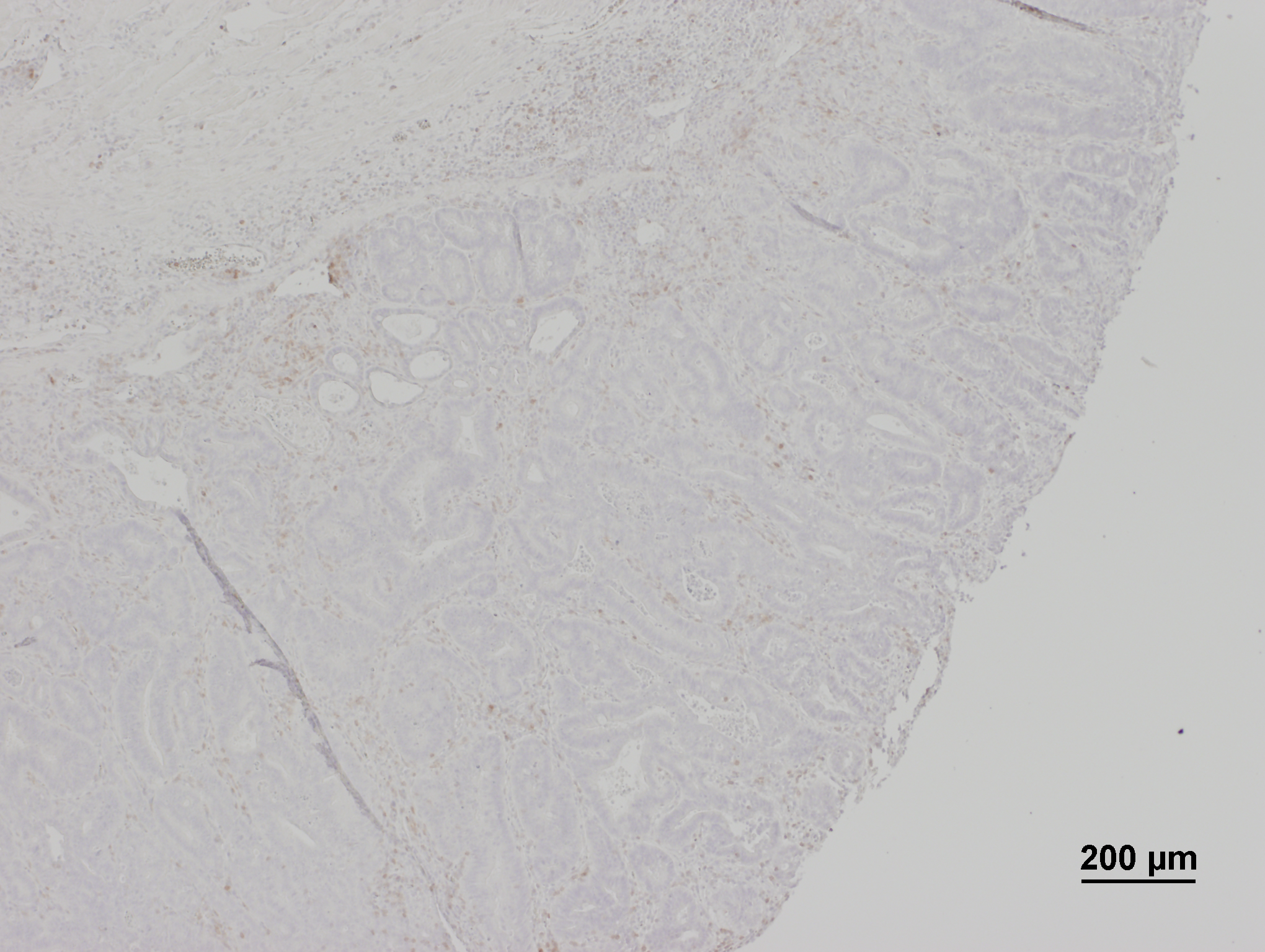

Supplement: Supplementary file 6 — Source Data for Figure 3 [file EMMM-12-e10681-s004.zip › fig3/Fig3Ei.tiff]

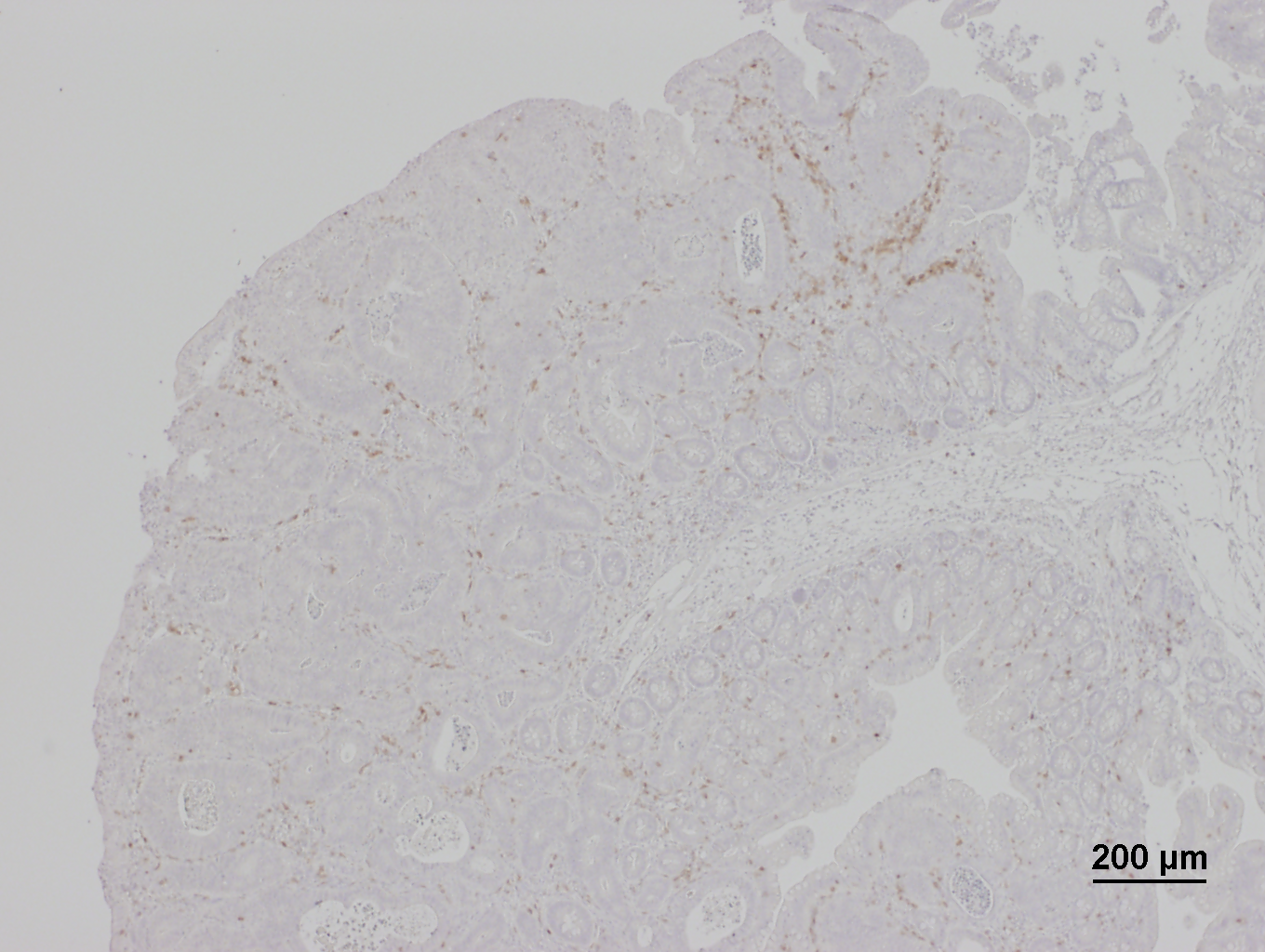

Supplement: Supplementary file 6 — Source Data for Figure 3 [file EMMM-12-e10681-s004.zip › fig3/Fig3Eii.tiff]

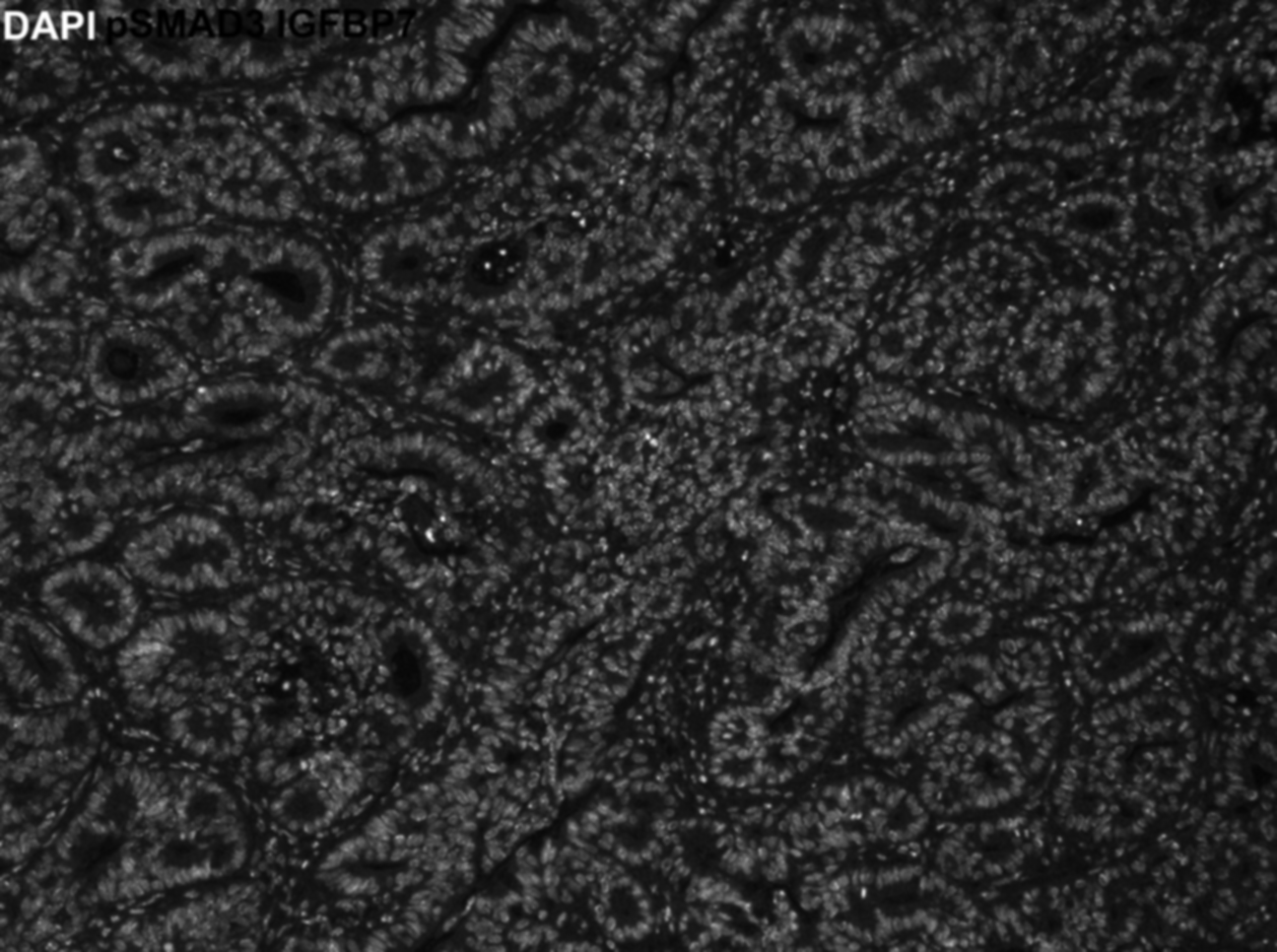

Supplement: Supplementary file 8 — Source Data for Figure 5 [file EMMM-12-e10681-s006.zip › Fig5/Fig5B_DAPI_pSMAD3_IGFBP7.tif]

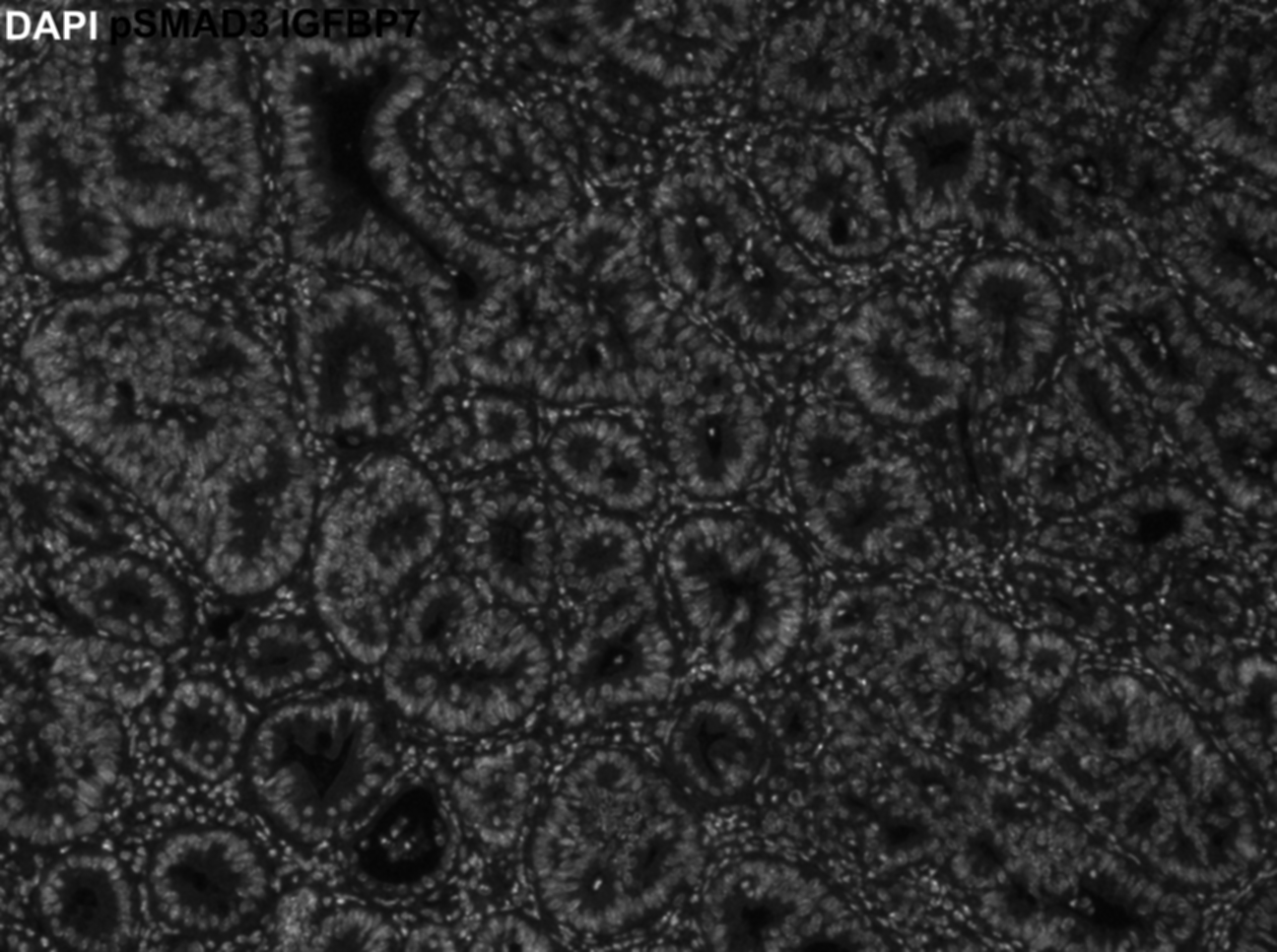

Supplement: Supplementary file 8 — Source Data for Figure 5 [file EMMM-12-e10681-s006.zip › Fig5/Fig5A_DAPI_pSMAD3_IGFBP7.tif]

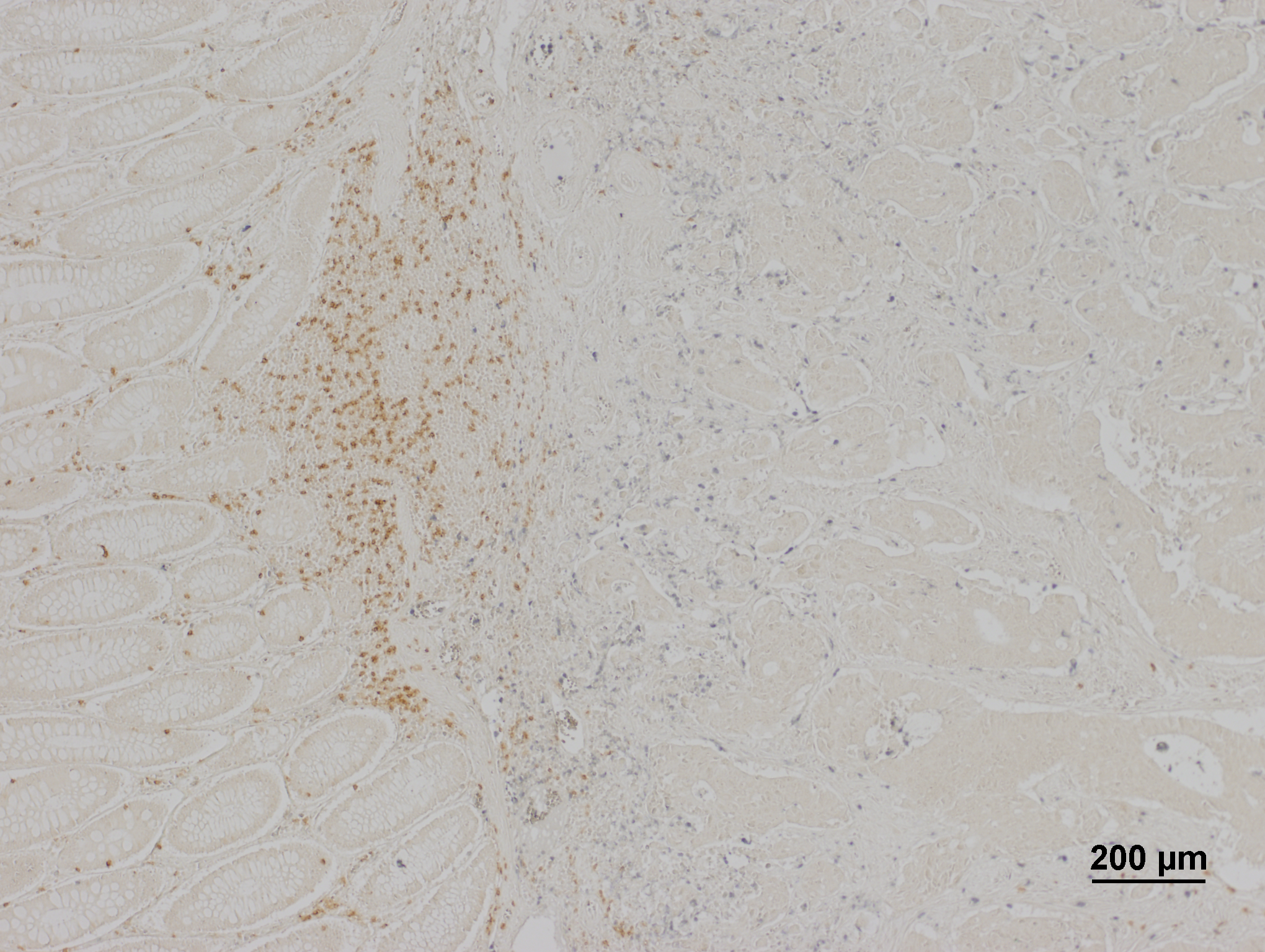

Supplement: Supplementary file 9 — Source Data for Figure 6 [file EMMM-12-e10681-s007.zip › fig6/Fig6B.tiff]

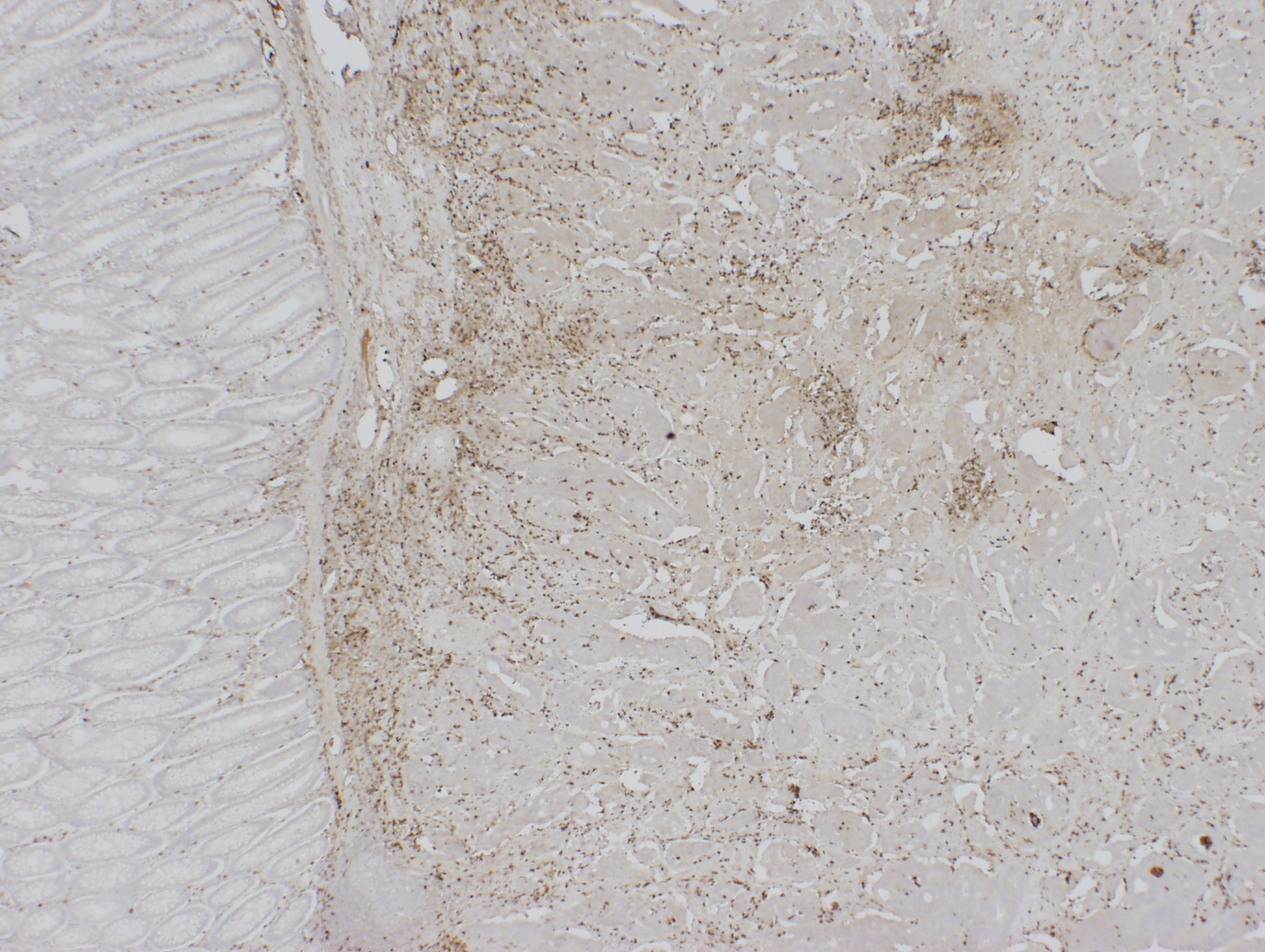

Supplement: Supplementary file 9 — Source Data for Figure 6 [file EMMM-12-e10681-s007.zip › fig6/Fig6A.jpg]
